# Substrate-Specific Differences in Catalytic Strategy and Activity across Ketol-Acid Reductoisomerase Variants

**DOI:** 10.1021/acs.jpcb.6c03868

**Published:** 2026-07-15

**Authors:** Elijah Karvelis, Bruce Tidor

**Affiliations:** † Department of Biological Engineering, 2167 Massachusetts Institute of Technology, Cambridge, Massachusetts 02139, United States; ‡ Computer Science and Artificial Intelligence Laboratory, Massachusetts Institute of Technology, Cambridge, Massachusetts 02139, United States; § Department of Electrical Engineering and Computer Science, Massachusetts Institute of Technology, Cambridge, Massachusetts 02139, United States

## Abstract

The specificity and catalytic efficiency of enzymes make them attractive for applications ranging from therapeutics to chemical manufacturing. However, it remains challenging to identify specific structural and dynamic mechanisms by which enzymes achieve their catalytic rate enhancements as well as to re-engineer enzymes to improve their catalytic properties. In earlier work we reported the design and selection of KARI mutants with calculated increases in specific activity (i.e., *k*_cat_) relative to wild type (WT) for the isomerization step of one of its native substrates: 2-acetolactate (ACL), which leads to the synthesis of the amino acids valine and leucine. Eight mutants were identified with computed improvements in *k*_cat_ of up to 4 orders of magnitude. In the current study, we investigate the effects of these same mutations on the isomerization of the other native substrate, 2-aceto-2-hydroxybutyrate (AHB, leading to the synthesis of isoleucine). Paralleling our previous work, we use the computational statistical mechanical method transition interface sampling (TIS) to simulate reaction kinetics and compute reaction rate constants. We find that the mutants selected for increased efficiency on ACL had varied levels of activity on AHB–some enhancing reactivity and others diminishing it–with the range in computed AHB rate constants spanning more than 7 orders of magnitude. Analysis of the simulations for WT-AHB revealed that only some of the structural mechanisms associated with mutants’ improved ACL catalysis were expected to transfer to, and thereby improve, AHB catalysis. For two mutants with significantly lower catalytic efficiency on AHB than WT, further analysis identified unique conformational changes that may explain their low activity on AHB.

## Graphical abstract

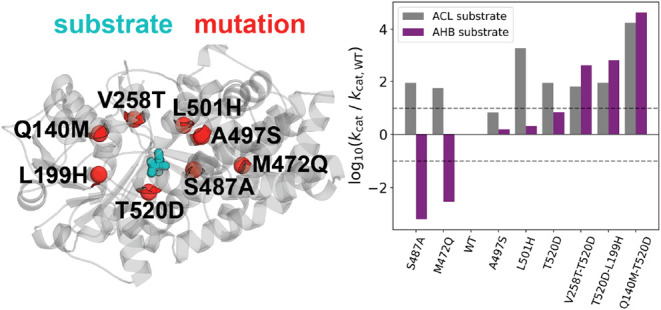

## Introduction

Enzymes can accelerate reactions by many orders of magnitude while maintaining high levels of specificity and selectivity. Despite the wide usage of enzymes across applications ranging from sustainable chemical production to therapeutics, our current understanding of the origins of enzymes’ catalytic power is limited.^1,2^ While the canonical interpretation of enzyme catalysis is couched in transition state theory (TST),^3^ other descriptions consider factors beyond transition state (TS) stabilization such as near-attack conformations,^4,5^ preorganization,^6^ promoting vibrations,^2^ or other interpretations involving the dynamic sampling of productive enzyme–substrate conformations primed for reacting.^7–11^ It is likely that enzyme rate enhancements are derived from some combination of these factors, underscoring the need for holistic approaches toward analyzing enzyme catalysis.

Computational path sampling techniques, such as transition interface sampling (TIS)^12^ and transition path sampling (TPS),^13,14^ are well-suited for studying enzyme catalysis, as they enable the unbiased sampling of ensembles of attempted reaction trajectories, which can then be used to analyze enzyme–substrate conformational dynamics before, during, and after attempted reactions.^7,8,15–23^ Additionally, statistical methods can be used to relate sampled transition path ensembles to reaction rates.^12–14^

Our group has previously used TPS and TIS to study ketol-acid reductoisomerase (KARI) from *Spinacia oleracea*,^7,8,24–26^ which catalyzes reactions for the synthesis of branched chain amino acids.^27,28^ When active, this homodimeric enzyme binds two Mg^2+^ ions, NADPH, and one of two substrates: (2*S*)-acetolactate (ACL) or (2*S*)-2-aceto-2-hydroxybutyrate (AHB) (Figure 1).^27–33^ Interestingly, KARI catalyzes two transformations of its substrate without the release of an intermediate, which include a rate-limiting isomerization involving either methyl (for ACL) or ethyl (for AHB) transfer and a subsequent NADPH-dependent reduction (Figure 2).^27–35^ Given its relevance to industrial-scale isobutanol production, several studies have engineered KARI variants with improved function. Most efforts focused on enhancing thermostability or shifting cofactor preference from NADPH to NADH,^36–39^ while others sought to improve specific activity.^24,40^ Complementing these engineering studies, systematic mutagenesis of active site residues has provided mechanistic insight into KARI catalysis, identifying residues such as D315 and E319 as important for catalytic function.^35,41^

**Figure 1:**
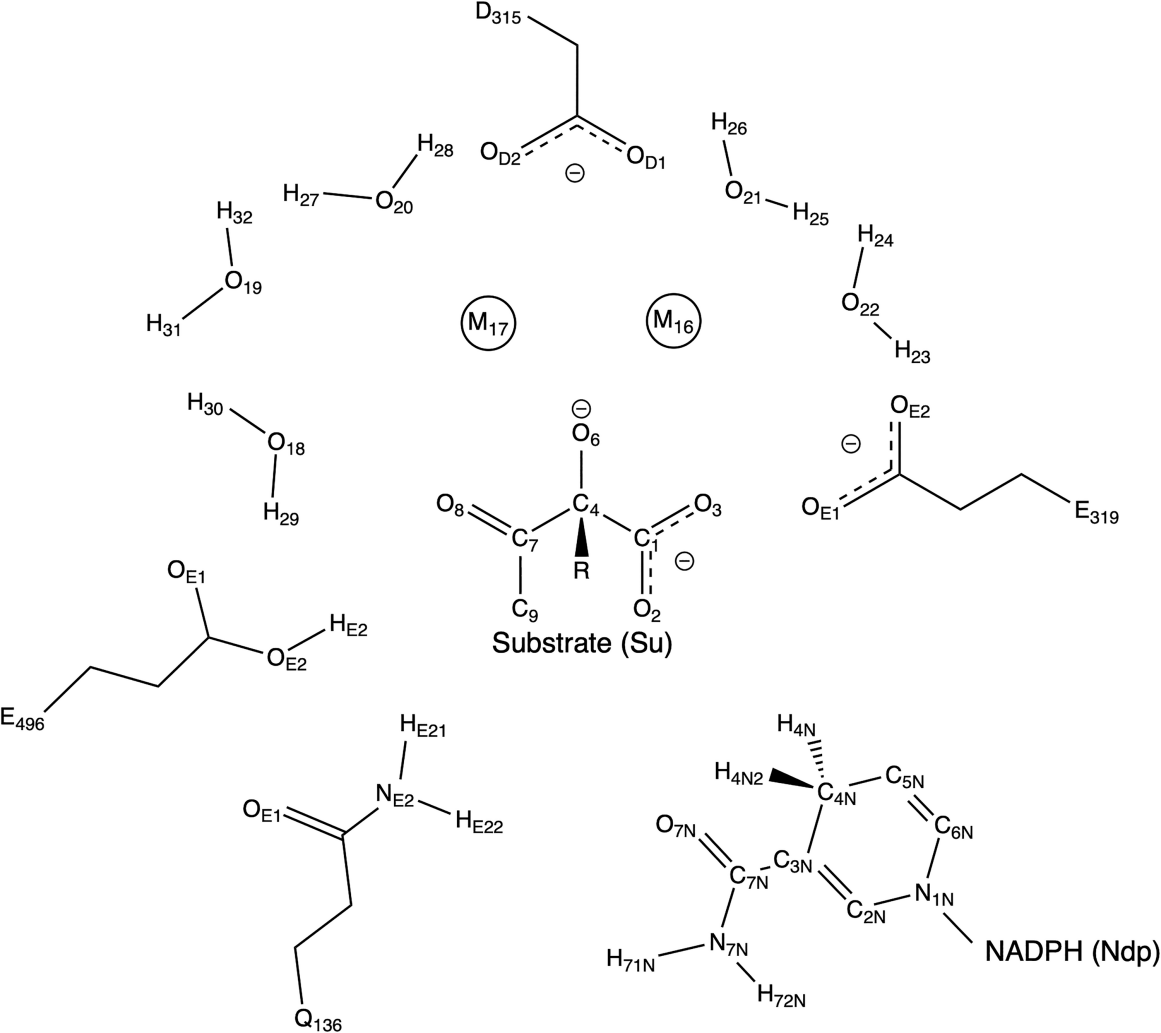
KARI active site. KARI binds two divalent Mg^2+^ cations (M_16_ and M_17_), NADPH, and one of two potential substrates: ACL (R = methyl) or AHB (R = ethyl). The Mg^2+^ ions are coordinated by waters, substrate, and catalytic residues D315, E319, and E496. Q136 can donate a hydrogen bond to the amide carbonyl oxygen (O_7*N*_) of NADPH’s nicotinamide. The configuration is shown for the reactant state immediately prior to the rate-limiting isomerization step. Atom naming conventions in this text use the group identifier MG6 when specifying Mg^2+^ ions or atoms belonging to their coordinating waters, and they use molecule identifier AC6 when specifying atoms in the substrate.

**Figure 2:**
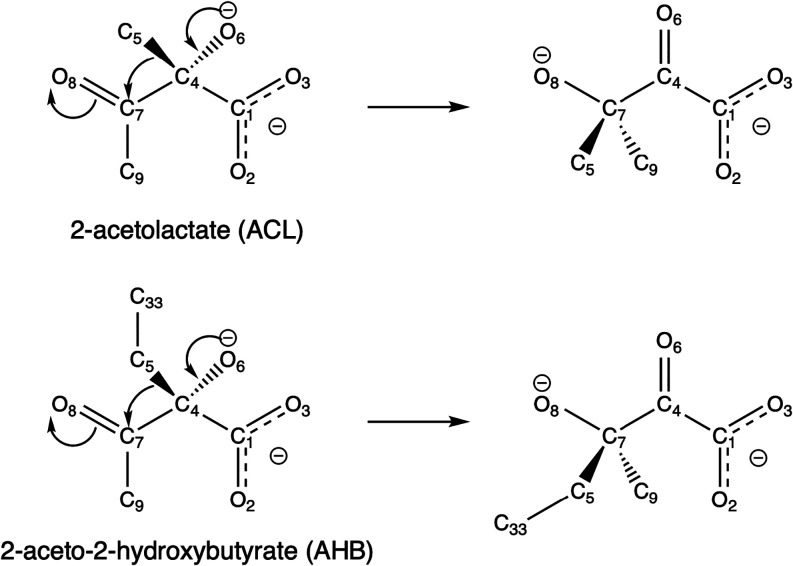
KARI-catalyzed isomerization of ACL and AHB. For simplicity of atom naming conventions, molecule identifier AC6 refers to either ACL or AHB throughout this text.

In previous studies of KARI-catalyzed ACL turnover, we used TIS and machine learning to identify a highly reactive subregion of the reactant well in wild-type (WT) KARI-ACL conformational space that, when populated, had a higher likelihood of reaching the product well than reaction attempts originating from conformers outside this region.^7^ In a follow-up study building on these findings, mutations with increased populations of such productive, reactive-like conformations were selected and shown to exhibit increased overall calculated specific activity compared to WT, and structural analyses of mutant and WT transition path ensembles identified three unique structural shifts associated with mutants’ increased reaction rates (*k*_cat_) on ACL: (i) the loss of a hydrogen bond from Q136 to NADPH, (ii) the perturbation of E319 conformation, and (iii) the increased adoption of eclipsed ACL conformations involving the migrating methyl group.^8^ Importantly, most mutants activated only two out of these three structural changes.

Increased specific activity on ACL may facilitate large-scale production of isobutanol, and for this engineering goal one may neglect KARI’s level of activity on its other native substrate, AHB. Nonetheless, characterization of AHB conversion is relevant for understanding KARI’s function and role in branched chain amino acid synthesis.^40,42–45^ In its native context, KARI typically maintains 5- to 8-fold higher *k*_cat_ on AHB than ACL, and the higher activity on AHB is believed to be under selective pressure for the maintenance of similar concentrations of isoleucine and valine.^28,46^ In this study, we investigate how the KARI mutations selected for increased activity on ACL affect AHB turnover. Using TIS, we computed reaction rate constants (*k*_cat_) for each KARI variant on AHB, uncovering a wide range in activities on AHB among mutants with increased activity on ACL. Combining the analysis of transition pathway ensembles with potential of mean force calculations, we show that the variations in activity on AHB can be largely explained by the nature of the structural changes implicated in increased catalytic efficiency on ACL. Specifically, the perturbed E319 conformation is found to associate with turnover efficiency of AHB as well as ACL, and the set of mutants activating this structural change tend to have increased activity on AHB relative to WT. The other two structural changes appear to selectively promote ACL turnover without necessarily having a similar effect on AHB catalysis. Collectively, these results highlight key similarities and differences in catalytic strategies on ACL and AHB, and they underscore the relation between prereaction enzyme–substrate conformational dynamics and turnover efficiency.

## Methods

### Structure Preparation and Equilibration

AHB-bound structures were prepared for each KARI variant starting from the corresponding minimized and equilibrated ACL-bound structure, which was prepared as described previously.^8^ In brief, CHARMM was used to build a migrating ethyl chain in place of the migrating methyl group in bound ACL. This used a newly added residue specifying the topology of AHB according to its optimized geometry as determined from ground state energy minimization implemented with GAUSSIAN03 ^47^ at the rhf/6–31g* level of QM theory. This geometry optimization also included nearby Mg^2+^ ions and Mg^2+^-coordinating waters. The AHB-bound structure was then energetically minimized in two stages using CHARMM. In the first stage, all atoms were held fixed except for hydrogens, and the structure underwent 100 steps of steepest descent minimization followed by 100 steps of adopted basis Newton–Raphson minimization. In the second minimization stage, AHB, Mg^2+^ ions, and coordinating oxygen atoms on D315, E319, and E496 were held fixed while all other atoms within 8 Å of AHB were harmonically constrained with a force constant of 50 kcal/(mol·Å^2^). The structure then underwent 10 rounds comprised of 100 steps of steepest descent minimization followed by 100 steps of adopted basis Newton–Raphson minimization, for which the harmonic constraints were reset before the start of each round. The minimized AHB-bound structure was then subjected to a 200 ps long equilibration MD run. This equilibrated structure was used for all downstream production simulations. All minimizations and dynamics simulations in CHARMM were executed using the setup described in Simulation Methodology section.

### Simulation Methodology

Energy minimizations and MD simulations were implemented with CHARMM^48,49^ version 39a1 with SQUANTUM, which included a custom modified RXNCOR module for TIS.^25^ A hybrid QM/MM treatment was executed using CHARMM’s SQUANTUM QM/MM module. To balance accuracy with computational efficiency, the QM region was treated with the semiempirical AM1 ^50^ QM force field, as done in previous studies.^7,8^ The QM region included AHB, Mg^2+^ ions and coordinating waters, the nicotinamide group of NADPH, and the side chains of residues D315, E319, and E496. Parameters for Mg^2+^ were taken from previous work by Stewart.^51^ The MM region was modeled by CHARMM36’s all-atom force field,^52^ and the Generalized Hybrid Orbital method^53^ treated the QM/MM boundary atoms: the α carbons of residues D315, E319, and E496 as well as the C_5_′ atom in NADPH. All molecular dynamics simulations were performed *in vacuo* with a distance dependent dielectric (1*r*). Langevin dynamics with a friction coefficient (*FBETA*) of 1 ps^–1^ was used to control temperature near 300 K. A 1-fs time step was used for all simulations.

### Seed Trajectory Generation

Starting reactive trajectories connecting the reactant well (state A, λ < −0.8 Å) to the product well (state B, λ > 0.8 Å) were generated by randomly choosing TS-like enzyme-AHB conformations from umbrella sampling simulations centered near λ ≈ 0 Å, removing their constraints, and running TIS shooting moves (see below). If a shooting move’s resulting trajectory connected the reactant and product states, then it was selected as a seed trajectory. Each seed trajectory was equilibrated by undergoing 2,000 additional TIS shooting moves. Equilibrated seed trajectories were used for initializing all downstream production TIS simulations.

### TIS Rate Constant Calculations

TIS rate constant calculations were executed according to the theory and procedures outlined by Van Erp et al.^12^ as previously described by Karvelis et al.^8^ To summarize, the calculation involved computing the flux factor Φ_*A*_ and the probability factor *P*(λ_B_|λ_A_). Here, λ_B_ indicates the interface at the “inner” edge of the product well, and λ_A_ = λ_1_ indicates the interface at the “outer” edge of the reactant well. The flux factor was calculated from 30 independent 400,000 fs-long QM/MM simulations by tracking the number of times the trajectory crossed λ_A_, having come from inside the reactant well, divided by total time inside the reactant well. The probability factor *P*(λ_B_|λ_A_) was computed as the product of 29 conditional probability terms, where each term reported the probability of reaching interface λ_*i*+1_ having reached interface λ_*i*_, such that *P*(λ_B_|λ_A_) = *P*(λ_B_|λ_*n*_)∏_*i*=1_^*n*–1^ *P*(λ_*i*+1_|λ_*i*_). Each term was calculated from an independent TIS ensemble. Interface ensembles were sampled for λ_*i*_ from −0.8 Å to 0 Å, in triplicate, starting from each of three unique seed trajectories. Interfaces between λ = −0.75 Å and λ = −0.15 Å were spaced 0.025 Å apart while the remaining interfaces were spaced 0.05 Å apart. A total of 6,000 shooting moves (described below) were attempted for each ensemble, and the first 3,000 moves were reserved for equilibration. This protocol provided nine independent *k*_cat_ values for each variant’s TIS rate constant calculation. The procedure and implementation were executed using a custom Python wrapper around CHARMM. That is, CHARMM was only used for running the individual dynamics simulations.

### TIS Shooting Moves

Shooting moves were implemented to sample new pathways and were performed as described previously.^8^ In brief, shooting moves followed the TIS algorithm described by Van Erp et al.,^12^ which was adapted with procedures outlined by Dellago et al.^54^ and Geissler and Chandler^55^ to additionally sample perturbations to kinetic energy such that the magnitudes of momenta displacements and kinetic energy changes could be separately controlled.

### Potential of Mean Force Calculations

Potential of Mean Force (PMF) calculations were used to compute free energy curves along the reaction order parameter, λ, as well as two-dimensional free energy surfaces along the order parameter and some other structural feature. The order parameter was defined as the difference in lengths of the breaking bond and the forming bond (λ = *dist*(AC6/C_5_,AC6/C_4_) – *dist*(AC6/C_5_,AC6/C_7_)). PMF profiles were computed using umbrella sampling with the weighted histogram analysis method (WHAM).^56^ Umbrella simulations were performed using CHARMM’s RXNCOR module to apply umbrella bias terms, and WHAM was implemented by software from Grossfield.^57^ For one-dimensional PMF curves, umbrella simulations were run for λ values spanning λ = −1.2 Å to λ = 1.2 Å, with consecutive windows placed 0.0325 Å apart in the range −0.5 Å < λ < 0.5 Å and 0.0975 Å apart outside this range. Each simulation used a force constant of 200 kcal/(mol·Å^2^) to harmonically restrain λ near the bias term minimum and was run for 100,000 steps with the first 50,000 reserved for equilibration. Three independent replicates were computed for one-dimensional PMF curves.

Two-dimensional PMF surfaces were calculated over λ, the first coordinate, and one of two other structural features as the secondary coordinate: (i) the dihedral angle over AHB/O_6_, AHB/C_4_, AHB/C_5_, AHB/C_33_ or (ii) the difference in the distances from MG6/M_16_ and MG6/M_17_ to MG6/O_21_ (i.e., *dist*(MG6/O_21_,MG6/M_16_) – *dist*(MG6/O_21_,MG6/M_17_)). These surfaces were computed from umbrella simulations that spanned a mesh grid over the two coordinates. The umbrella term values along λ were placed as previously described for the one-dimensional PMF curves along λ. For secondary coordinate (i), umbrella term values were placed from −175° to 175° with 10° spacing from −85° to 85° and 15° spacing outside this range. This coordinate was harmonically restrained with a period of 2π radians and a force constant of 20 kcal/(mol·rad^2^). For secondary coordinate (ii), umbrella term values were placed from −3.0 Å to 2.5 Å with 0.1 Å spacing from −1.0 Å to 1.0 Å and 0.25 Å spacing outside this range. A force constant of 20 kcal/(mol·Å^2^) was used to harmonically restrain this secondary coordinate. For two-dimensional umbrella simulations with a λ term minimum greater than −1.0 Å, the λ coordinate was gradually pulled into place by a series of 1,000 fs-long simulations that increased the λ term minimum from −1.0 Å to the desired minimum placement by increments of 0.1 Å. These incremental simulations also applied the umbrella term corresponding to the secondary coordinate. For all pairwise combinations of λ minima and secondary coordinate minima, an umbrella simulation with the corresponding restraints was run for 50,000 fs with the first 25,000 fs reserved for equilibration, which was shown to be long enough for the computed free energy surface to converge.

### Structural Feature Extraction and Logistic Regression

Ten independent ensembles were collected for both reactive trajectories (R) and nonreactive pathways that reached λ > −0.4 Å (NR_–0.4_). Each ensemble was generated from 4,000 shooting moves, and structural features were computed at each time step in accepted simulations. These features included the 70 interatomic distances, angles, and torsions used previously^8^ with the addition of two dihedral angles describing the conformation of AHB’s migrating ethyl: (i) the torsion along O_6_, C_4_, C_5_, and C_33_ and (ii) the minimum torsion along C_4_, C_5_, C_33_, and any one of C_33_’s bound hydrogen atoms. Individual simulations were time-aligned by defining *t* = 0 at the last compression of the breaking bond, as described previously;^8^ 300 1-fs time steps were included prior to *t* = 0, and 200 1-fs time steps were included following *t* = 0 to provide dynamical information before, during, and after the attempted reaction event. Data were compiled across different KARI variants into a single data set by sampling 500 accepted pathways per ensemble according to TIS path counts. This procedure gave equal representation to R and NR_–0.4_ paths and all KARI variants. A similar data set using the original 70 features was reconstructed for KARI-ACL. These data sets were used for all analyses reporting on specific structural features. Using these data, logistic regression models were trained to classify time-averaged WT-AHB conformations over −160 to −130 fs as either R or NR_–0.4_, and LASSO regression was used to select the most optimal 15-feature subset for this task.^58^ These models were trained on 90% of the data with the other 10% reserved for testing. Model performance was reported for models retrained on the selected feature subset without regularization.

## Results and Discussion

### WT Exhibits Substrate-Specific Differences in Catalytic Strategy and Overall Activity

The reaction rate constant for WT-AHB (*k*_cat,AHB_) was computed to be (2 ± 1) × 10^–14^ s^–1^, 200-fold larger than that calculated for WT-ACL (*k*_cat,ACL_). The calculated increase in WT’s activity on AHB relative to ACL corroborated experimental observations of 5- to 8-fold faster.^28,46^ The flux factor, or rate at which reactions were attempted, was about 7-fold lower for WT-AHB than WT-ACL. Thus, the enhanced activity on AHB relative to ACL was driven by an increased reaction probability; that is, attempted reactions were more likely to be successful for AHB than for ACL. The TIS rate calculations were composed of conditional probabilities describing the likelihood that the enzyme–substrate complex would continue δ units along the order parameter λ (i.e., toward the product well), as opposed to returning to the reactant well, given that it had already reached some level of progress along λ

1 P(λ+δ|λ)

where, as in prior work,^7,8^ λ was defined as the length of the bond being broken (distance between C_4_ and C_5_) minus the length of the bond being formed (distance between C_7_ and C_5_) such that λ < −0.8 Å defined the reactant well and λ > 0.8 Å defined the product well. These conditional probabilities report the catalytic efficiency at different points along the reaction, while their product indicates the cumulative probability of the enzyme–substrate complex reaching some level of progress along λ having started from the outer edge of the reactant well (λ_A_ = −0.8 Å)

2 $$P\left(\lambda |\lambda_{A}\right)=P\left(\lambda |\lambda_{n}\right)\prod_{i=1}^{n-1}P\left(\lambda_{i+1}|\lambda_{i}\right)$$

where λ_1_ = λ_A_ and *n* is the number of TIS interfaces preceding λ. We compared the conditional, local probability terms (Figure 3A) and cumulative probabilities (Figure 3B) to compare the efficiencies of different parts of the reaction for ACL and AHB. The local probability of further incremental reaction progress along the order parameter λ indicated similar probability profiles for both substrates during the first half of the reaction but consistently larger probabilities for AHB than ACL during the second half of the reaction (Figure 3A). The minima along these probability profiles indicate the points along the reaction at which the enzyme–substrate complex is most likely to revert back to the reactant well, which is here referred to as the “kinetic bottleneck.” Notably, the kinetic bottleneck for AHB occurs at an earlier point along the order parameter λ and has higher probability of continuing the reaction than the kinetic bottleneck for ACL (Figure 3A).

**Figure 3:**
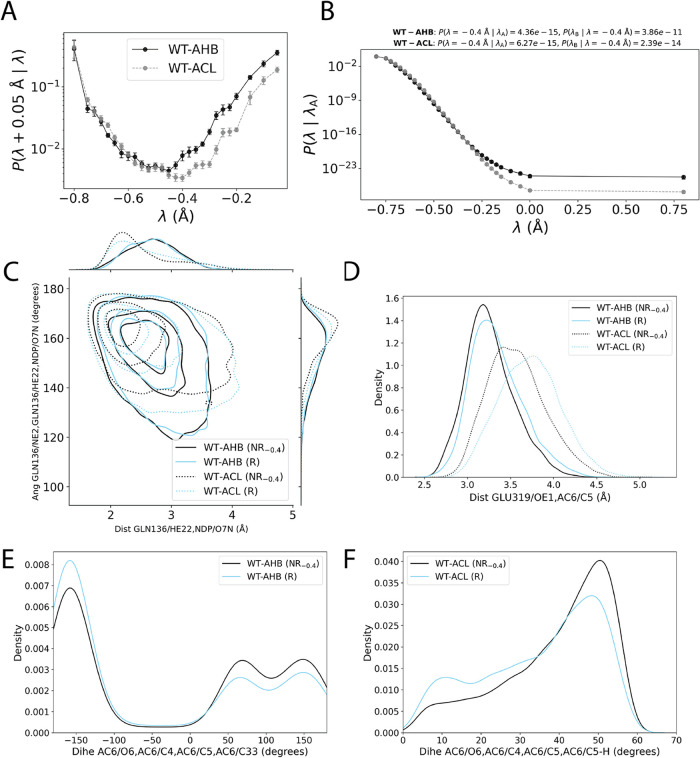
Characterization of WT-AHB catalysis. (A) “Kinetic probability plots”–the local probability of an attempted reaction making incrementally further progress along λ given that a certain level of progress was already made (*P*(λ + 0.05 Å|λ)), shown for WT-AHB (black) and WT-ACL (gray). Circle markers indicate the average and error bars designate ± SEM (*n* = 9 independent TIS rate calculations). (B) Cumulative kinetic probability of the reaction complex reaching a certain level of reaction progress, given that the complex began in the reactant well (*P*(λ|λ_A_)), shown for WT-AHB (black) and WT-ACL (gray). The region where λ < −0.8 Å = λ_A_ is considered the reactant well and is referred to as state A. The region in which λ > 0.8 Å = λ_B_ is considered the product well and is referred to as state B. Circle markers indicate the average and error bars designate ± SEM (*n* = 9 independent TIS rate calculations). (C–F) Distributions of structural features within the −160 to −130 fs time window for reactive (R, blue) and nonreactive (NR_–0.4_, black) trajectories sampled by WT-ACL (dashed) and WT-AHB (solid). (C) Q136–NADPH hydrogen bond distance and angle. (D) Distance from E319 carboxylate oxygen to migrating methyl (C_5_). (E) Dihedral angle describing AHB ethyl conformation. (F) Dihedral angle describing ACL methyl conformation. Panels (A–D, F) include data reproduced from ref 8.

An earlier study by our group identified three structural changes associated with increased catalytic efficiency (i.e., a higher frequency of occurrence in successful reaction attempts than failed ones) in WT-ACL and increased overall activity in several mutants on ACL: (i) the loss of a hydrogen bond from Q136 to NADPH, (ii) the perturbation of E319 conformation, and (iii) the increased adoption of eclipsed substrate conformations involving the migrating methyl group.^8^ To determine whether these same structural changes were associated with increased catalytic efficiency for AHB, geometric features describing these structural changes were tracked in WT-AHB simulations of reactive (R) and nonreactive trajectories making it at least to λ = −0.4 Å before returning to the reactant well where λ < −0.8 Å (NR_–0.4_).

Structural change (i), the loss of the hydrogen bond from Q136 to NADPH, was associated with successful turnover of ACL as indicated by the shift in the WT-ACL (R) distribution relative to the WT-ACL (NR_–0.4_) distribution, which reflected that WT-ACL complexes without the hydrogen bond were more likely to successfully react (Figure 3C). WT-AHB behaved differently. Here, both the WT-AHB R and NR_–0.4_ distributions were shifted toward less favorable Q136–NADPH hydrogen bonding geometries than measured for WT-ACL trajectories. Furthermore, the WT-AHB R and NR_–0.4_ distributions closely overlapped (Figure 3C). In summary, while the loss of the hydrogen bond was associated with R paths for WT-ACL, no such correlation existed for WT-AHB because both its NR_–0.4_ and R paths were similarly characterized by suboptimal Q136–NADPH hydrogen bonding geometries.

Structural change (ii), which involved an adjusted E319 conformation, affected WT-AHB catalytic efficiency similar to how it had WT-ACL. Specifically, for WT-ACL, R paths were associated with increased distances between the E319 carboxylate and the migrating methyl, which could be accessed upon rotation of the E319 carboxylate (Figure 3D). Both the WT-AHB R and NR_–0.4_ distributions were shifted toward decreased distances relative to WT-ACL, and they similarly suggested that larger distances, perhaps due to rotation of the E319 carboxylate (Figure S3), were associated with successful turnover of AHB as indicated by the rightward shift of WT-AHB R relative to WT-AHB NR_–0.4_ trajectories (Figure 3D).

Structural change (iii) involved the increased adoption of substrate conformations with an eclipsed migrating methyl. This change was not directly comparable between WT-ACL and WT-AHB due to the change in identity of the migrating group, so we consider the corresponding WT-AHB migrating ethyl conformation (i.e., the dihedral describing its torsional orientation, Figure 3E) separately from the WT-ACL migrating methyl conformation (Figure 3F). Near-eclipsed conformations were associated with successful turnover (R trajectories) for WT-ACL as indicated by the increased relative density of R pathways compared to NR_–0.4_ ones in this region (Figure 3F). WT-AHB, however, lacked a clear relation between eclipsed conformations and reaction efficiency, as indicated by variable differences between WT-AHB R and WT-AHB NR_–0.4_ distributions across the three eclipsed conformations: little difference at 0°, a slight bias for R trajectories at −120°, and a bias for NR_–0.4_ trajectories at 120° (Figure 3E). Staggered conformations were associated with failed turnover (NR_–0.4_ trajectories) for WT-ACL as indicated by the increased relative density of NR_–0.4_ pathways compared to R ones for torsional orientations greater than 30° (Figure 3F). The association between catalytic efficiency and WT-AHB staggered conformations was more nuanced. Nearly anti conformations around −150° to −170° were most strongly associated with R trajectories relative to NR_–0.4_ ones, suggesting that this staggered conformation was especially conducive to AHB turnover. The gauche conformation at 60°, however, was associated with NR_–0.4_ attempts. Critically, the gauche conformation around −60° was rarely sampled, perhaps suggestive of its low stability or poor priming for turnover.

Logistic LASSO regression models supported these observations. Here, models were trained to classify between WT-AHB R and NR_–0.4_ pathways’ time-averaged conformations over the early time window of −160 to −130 fs, where *t* = 0 fs was defined at the last compression of the potentially breaking bond between C_4_ and C_5_ as done in prior studies.^7,8^ LASSO regression was used to select the most predictive subset of 15 features.^58^ The test accuracy and AUROC were 0.713 and 0.766, respectively, and the selected features reported on E319 conformation (distance from E319/O_E1_ to AHB/C_5_) and migrating ethyl conformation (dihedral along AHB/O_6_, AHB/C_4_, AHB/C_5_, and AHB/C_33_) among other features describing the conformation of substrate and cofactors. None of the selected features were directly related to the Q136–NADPH hydrogen bond. While the selected distance from AHB/O_2_ to NDP/N_7N_ included the NADPH amide group involved in the Q136–NADPH hydrogen bond, this distance was not as strongly correlated with the strength (i.e., length) of the hydrogen bond in WT-AHB (0.23 Pearson’s correlation coefficient) as it was in WT-ACL (0.65 Pearson’s correlation coefficient). To summarize, the selected feature set indicated that both E319 and migrating alkyl conformations were associated with turnover of AHB, similar to ACL. However, the hydrogen bond from Q136 to NADPH, which was relevant to catalysis of ACL, was not identified as catalytically important for conversion of AHB.

### Enzyme Mutations Differentially Affect Activity on Two Native Substrates

Rate constants for the KARI-catalyzed alkyl transfer step (Figure 2) on ACL and AHB were computed for each enzyme variant using TIS rate calculations. While each of the eight mutants was previously shown to have significantly increased computed specific activity on ACL relative to WT, only three of these mutants had substantially larger computed specific activity (i.e., a greater than 10-fold increase) on AHB than WT: Q140M-T520D, T520D-L199H, and V258T-T520D. The AHB rate constants computed for three of the other mutants—T520D, L501H, and A497S—were comparable to WT’s (i.e., within 10-fold). Interestingly, the AHB rate constants calculated for the final two mutants—M472Q and S487A—were each more than 100-fold lower than WT’s, which indicated that these mutants were computed to have selectively facilitated ACL’s methyl transfer but hindered AHB’s ethyl transfer (Figure 4).

**Figure 4:**
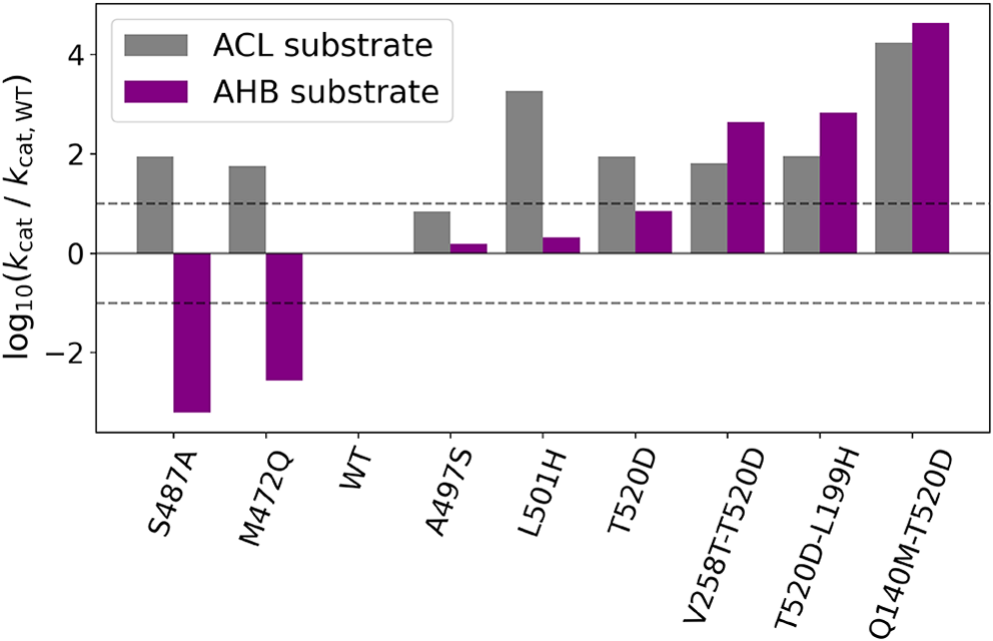
TIS-computed rate constants for AHB (purple) and ACL (gray) isomerization by KARI enzyme variants (values listed in Table S1). Bars indicate the base-10 logarithm of the ratio of mutant to WT *k*_cat_ (averaged over *n* = 9 independent TIS rate calculations). Dashed lines indicate 10-fold difference in computed *k*_cat_ relative to WT. Values for ACL were reproduced from ref 8.

Collectively, these results indicate that while some (two out of eight) of the mutants designed for increased activity on ACL had much lower computed activity on AHB, this trade-off was often not the case, with three mutants maintaining WT-like computed activity on AHB and another three mutants actually enhancing computed activity on AHB. In particular, the four T520D-containing mutants maintained or exceeded WT-like activity on AHB, which suggested that the T520D mutation induced interactions favorable for both ACL’s and AHB’s alkyl transfer. A497S and L501H, with AHB rate constants only 2-fold greater than WT’s, likely increased activity on ACL by introducing structural and dynamic changes that neither negatively impacted nor helped catalysis of AHB. S487A and M472Q, with large differences in relative activities on ACL and AHB, drove conformational changes that were conducive to turnover of ACL but detrimental to that of AHB. All three cases present interesting behavior worthy of further study and mechanistic investigation.

### Kinetic Profiles Indicate Variability in Reaction Efficiencies across Enzyme Variants

The TIS flux factor, or rate of departing the reactant well, was not the dominating factor in computed differences in *k*_cat_ across KARI variants (Table S2); reaction probability was. Thus, we compared conditional probability terms (Figure 5) and cumulative probabilities (Figure S1) from TIS rate constant calculations to determine which parts of the reaction were made more or less efficient by different mutations. The three mutants with large increases (i.e., greater than 10-fold) in computed *k*_cat_ for AHB: Q140M-T520D, T520D-L199H, and V258T-T520D—were actually less efficient than WT from the reactant well (λ_A_ = −0.8 Å) to around λ = −0.5 Å, which is about halfway up the reaction barrier. Their catalytic improvements on AHB occurred along the remainder of the path to the top of the barrier (from λ = −0.5 Å to λ = 0 Å), and these enhancements more than offset the decreased efficiency during the initial climb of the reaction barrier such that these mutants were more catalytically efficient than WT overall (Figures 5 and S1, left columns). The decreased efficiencies of T520D-containing double mutants relative to WT during the first half of AHB isomerization contrasts with observations of ACL turnover, where the T520D-containing double mutants’ conditional probability terms were generally comparable to or greater than WT’s along the full profile of the reaction (Figure 5, right column).

**Figure 5:**
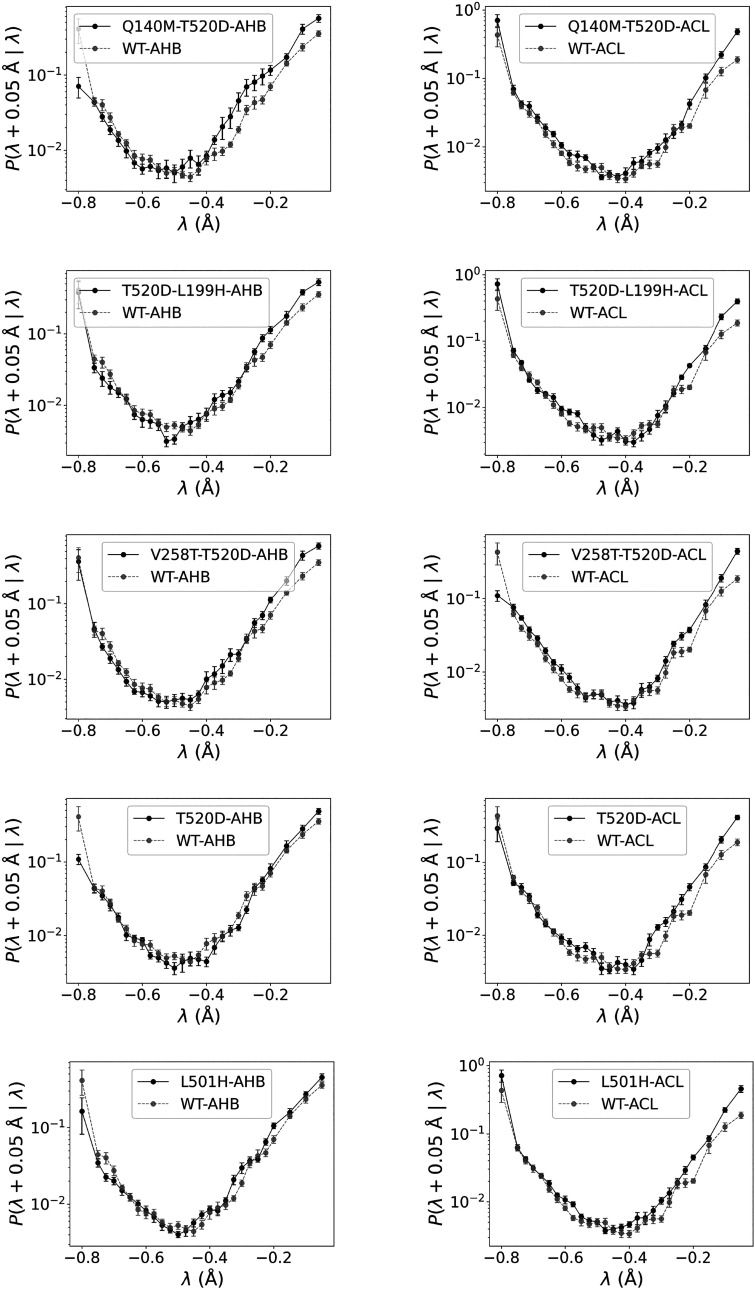

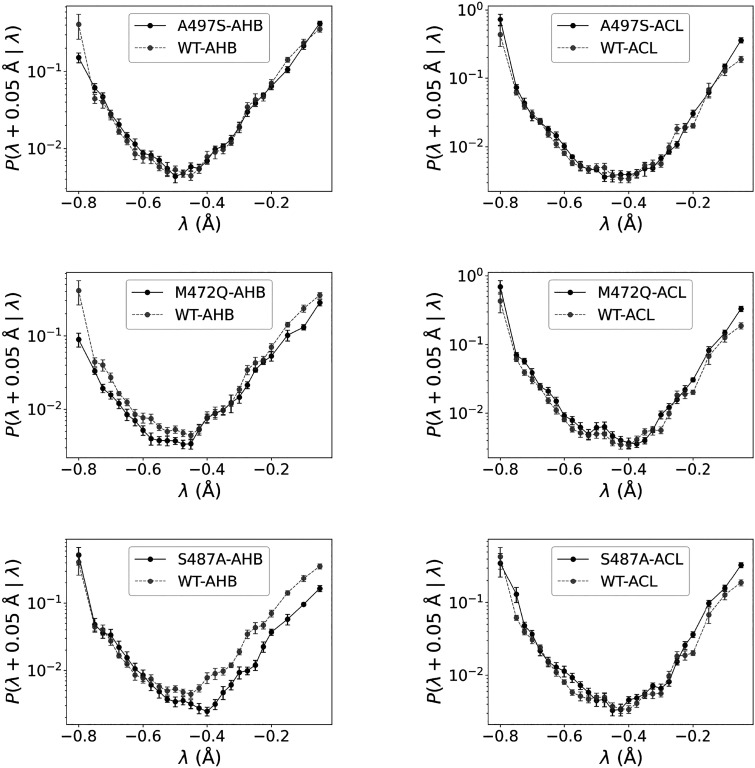
Kinetic probability plots as previously described shown here for mutant (black) and WT (gray) catalysis of AHB (left) and ACL (right). ACL data were reproduced from ref 8.

The three mutants with comparable AHB activity to WT were T520D, L501H, and A497S. Their conditional (Figure 5, left column) and cumulative (Figure S1, left column) probabilities for AHB isomerization were mostly consistent with those computed for WT along the path from the reactant well at λ = −0.8 Å to the top of the barrier near λ = 0 Å. That is, none of these three mutants significantly altered the profile of AHB reaction progress efficiency. This contrasts with observations of ACL catalysis by these mutants, where all three mutants’ conditional probability terms were mostly comparable to or larger than WT’s throughout the reaction profile, and T520D and L501H both exhibited especially increased efficiencies relative to WT during the second half of the reaction following the kinetic bottleneck (Figure 5, right column).

M472Q and S487A each had computed *k*_cat_ more than 100-fold lower than WT for AHB, but their conditional (Figure 5, left column) and cumulative (Figure S1, left column) probabilities indicated significant differences in their catalytic efficiencies at different points along the reaction. In particular, M472Q-AHB was consistently less efficient than WT-AHB, but its difference was especially pronounced along the initial climb of the reaction barrier from λ_A_ = −0.8 Å to about λ = −0.45 Å. In contrast, S487A-AHB maintained progress efficiencies that were comparable to WT-AHB’s for most of the initial part of the reaction (from λ_A_ = −0.8 Å to λ = −0.6 Å) but were significantly smaller along the remainder of the path to the top of the barrier. Furthermore, the lowest conditional probability along the reaction was lower for S487A-AHB than for WT-AHB by 1.8-fold, which indicated a tighter kinetic bottleneck for S487A-AHB than for WT-AHB. For ACL isomerization, both M472Q and S487A were computed to have conditional reaction probability terms that were roughly equal to or larger than WT’s across the full profile of the reaction (Figure 5, right column).

### Distinct Catalytic Strategies Identified across Different Enzyme Variants

We next characterized the catalytic strategies employed by different enzyme variants when catalyzing the isomerization (ethyl transfer) of AHB. Here, the general approach was to compare the structural and dynamic characteristics of the active site across different enzyme variants in both R and NR_–0.4_ trajectories. TIS was used to sample R and NR_–0.4_ pathway ensembles. From each of 10 independent ensembles collected for each pathway type, 500 representative trajectories were sampled (weighted by pathway count). For each pathway, 72 geometric features including interatomic distances, angles, and dihedral angles were computed at every time point to describe the structure of the enzyme-AHB active site. These 72 features included the 70 described in Karvelis et al.^8^ with the addition of two dihedral angles specific to the conformation of the migrating ethyl group: (i) the torsion along O_6_, C_4_, C_5_, and C_33_ and (ii) the minimum torsion along C_4_, C_5_, C_33_, and any one of C_33_’s bound hydrogen atoms. For comparison, a similar data set of equal size, but using only the original 70 features, was constructed for the catalysis of ACL.

To visualize the R and NR_–0.4_ pathways across all AHB-bound enzyme variants, we projected the data into two dimensions using UMAP.^59^ The plot of the embedded data (Figure 6) shows a number of interesting features. A variety of conformations are sampled in the prereactive window across all of the mutants. The distributions of reactive and nonreactive conformations overlap in their projection. Moreover, each mutant tends to adopt a few conformational clusters that are both distinct from each other and from those of the other mutants. The exception is the set of T520D-containing mutants, whose conformations overlap each other in these projections, consistent with a picture in which a second mutation’s influence on conformation is insufficient to significantly overcome the dominance of T520D.

**Figure 6:**
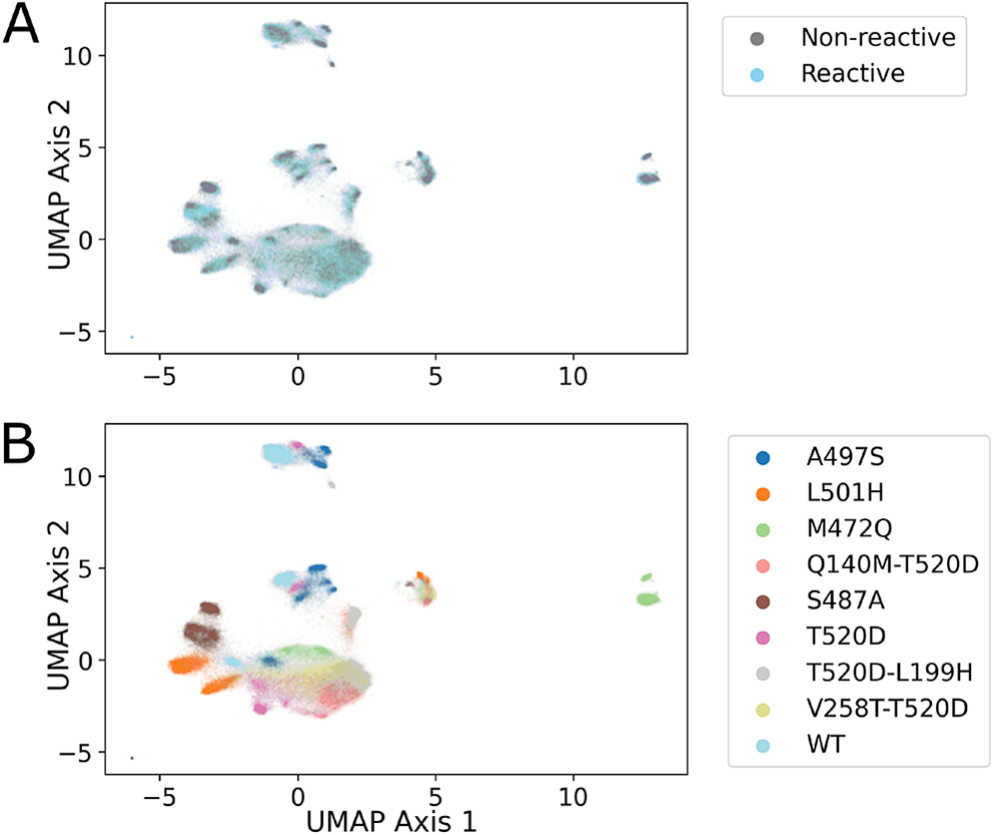
UMAP embedding of structural features describing enzyme–substrate complexes from KARI-AHB trajectories colored by (A) pathway type or (B) enzyme variant. The embedded conformations were randomly sampled from the −160 to −130 fs time window, and the two-dimensional UMAP embedding was constructed using the Euclidean distance metric with 15 nearest neighbors and a minimum distance of 0.1.

### T520D Mutation Influenced E319 Conformation

The four T520D-containing mutants were embedded similarly by UMAP and overlapped within −2 < UMAP Axis 1 < 3 and −3 < UMAP Axis 2 < 0. This region in the embedding was largely devoid of other variants’ data, which suggested the presence of structural characteristics unique to the T520D-containing mutants (Figures 6 and S2). Our previous analysis of ACL turnover identified the mechanism through which the T520D mutation facilitated ACL catalysis as a rotation of the E319 carboxylate and increase in its distance from the substrate’s migrating group (C_5_).^8^ This rotation more closely aligned the axis joining the E319 carboxylate oxygens with the axis joining C_4_ and O_6_ in the substrate (Figure 7B). The rotated E319 conformation, and concomitant increase in distance from E319 carboxylate to C_5_, was again observed for T520D-containing mutants catalyzing AHB (Figures 7A and S3). This conformation was not observed for any other enzyme variants (Figure S3). Similar to observations on ACL, the increased E319 carboxylate to C_5_ distance was associated with successful catalysis because R pathways were right-shifted relative to NR_–0.4_ ones (Figure 7A). Furthermore, the observation that three of the four T520D-containing mutants exhibited greater than 100-fold increases in computed activity on AHB relative to WT supports the notion that the rotated E319 conformation was beneficial for catalysis. The fourth mutant, T520D single mutant, had a 7-fold increased computed activity on AHB relative to WT. The source of T520D single mutant’s decreased efficiency on AHB relative to the T520D-containing double mutants is not clear.

**Figure 7:**
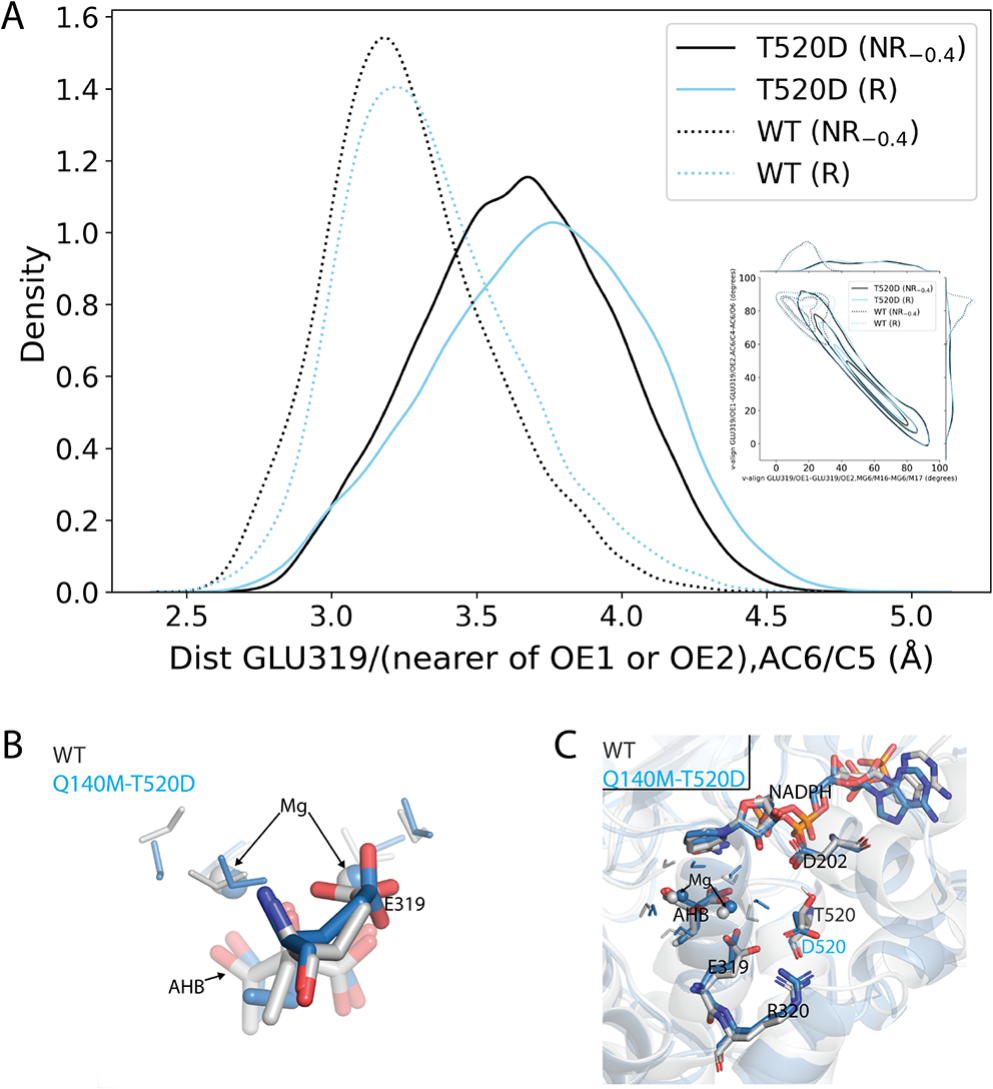
Catalysis of AHB by T520D-containing mutants. (A) Distributions for the distance from the methylene in the migrating ethyl (C_5_) to the nearest E319 carboxylate oxygen atom (O_E1_ or O_E2_) for WT (dashed) and T520D-containing mutants (solid) R (blue) and NR_–0.4_ (black) pathways. The lengthened distance in T520D-containing mutants was caused by a rotation of the E319 carboxylate in which the axis joining the carboxylate oxygens more closely aligned with the axis joining AHB atoms C_4_ and O_6_ than the axis joining the Mg^2+^ cofactors (inset, see Figure S3). (B) The rotated E319 conformation sampled by T520D-containing mutants shown for Q140M-T520D (blue) overlaid with the WT-like conformation shown for WT (gray). (C) Overlay of WT (gray) and Q140M-T520D (blue) showing the participation of residue 520 in a network of electrostatic interactions involving other active site residues, substrate, cofactors, and waters.

As observed in enzyme-ACL trajectories, residue 520 participated together with E319 in a network of hydrogen bonds and electrostatic interactions in the active site. In WT, T520 donated a hydrogen bond to D202 and pointed away from E319. The mutated D520 residue instead formed a salt bridge with R320, which stabilized D520's negatively charged carboxylate in a position pointing toward E319, and the resulting electrostatic repulsion likely contributed to E319’s adoption of its rotated conformation (Figure 7C).

Collectively, these results indicate that the T520D mutation caused a rotated E319 conformation not observed for WT, which was associated with successful conversion of AHB, not just ACL. Hence, it is likely that the T520D-containing mutants tended to increase activity on AHB because the mechanism by which they reached increased catalytic efficiency on ACL also applied to AHB turnover.

### L501H Selectively Increased Activity on ACL, But Not AHB, Relative to WT

Our previous investigation suggested that L501H attained increased catalytic efficiency on ACL compared to WT by weakening the hydrogen bond from Q136 to NADPH and increasing the frequency with which eclipsed conformations involving the migrating methyl were sampled.^8^ In contrast to ACL, simulations of WT-AHB R and NR_–0.4_ trajectories indicated similar Q136–NADPH hydrogen bonding in successful and failed reaction attempts, suggesting that L501H-AHB could not gain a catalytic advantage over WT by way of weakening the Q136–NADPH hydrogen bond. Furthermore, the Q136–NADPH hydrogen bond features were similar for L501H-AHB and WT-AHB, with both the R and NR_–0.4_ distributions significantly overlapping for both variants (Figure 8A). Therefore, L501H did not acquire a catalytic advantage over WT for AHB turnover by further weakening the Q136–NADPH hydrogen bond. With regard to the migrating ethyl conformation, WT-AHB was most efficient when nearly antistaggered on the negative side, but it also underwent reaction attempts from other gauche and eclipsed conformations (though not all of them). L501H-AHB was restricted to only the gauche conformation near 60°, and it never sampled the nearly antistaggered conformation found to be most efficient for WT-AHB. Therefore, it is unlikely that L501H-AHB could gain an increase in catalytic efficiency relative to WT-AHB due to the conformational sampling of the migrating ethyl.

**Figure 8:**
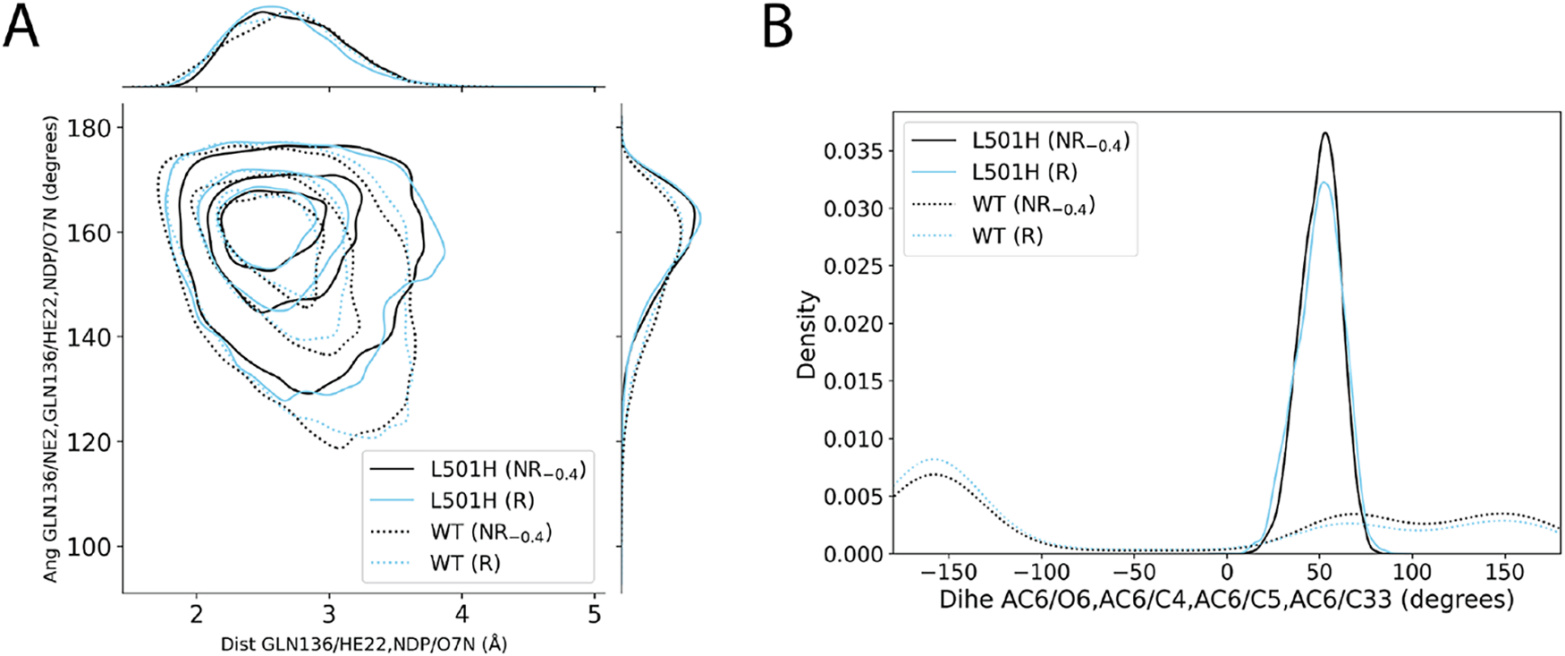
L501H-AHB catalysis. Distributions of structural features within the −160 to −130 fs time window for reactive (R, blue) and nonreactive (NR_–0.4_, black) trajectories sampled by WT-AHB (dashed) and L501H-AHB (solid). (A) Q136–NADPH hydrogen bond distance and angle distributions. (B) Dihedral angle distributions for AHB ethyl conformation.

Hence, while the loss of the Q136–NADPH hydrogen bond and increased eclipsing of substrate were conducive to ACL turnover, it appears that these mechanisms do not necessarily transfer to AHB turnover. Indeed, the three mutants with increased activity relative to WT on ACL through these two structural mechanisms did not show increased activity on AHB: S487A (slower-than-WT), M472Q (slower-than-WT), and L501H (neutral).

### Migrating Ethyl Conformation Linked to S487A-AHB Catalytic Efficiency

S487A was computed to have increased activity on ACL but decreased activity on AHB relative to WT (Figure 4). This result suggests that S487A’s increased activity on ACL was driven by a mechanism that did not apply to the catalysis of AHB, either because the AHB reactive complex did not adopt the same conformation as the ACL complex or because it did adopt the same conformation but did not gain the catalytic benefit (keeping in mind that the change in substrate replaces the migrating methyl of ACL with a migrating ethyl). Our earlier analysis attributed S487A’s enhanced activity on ACL relative to WT to (i) weakening of the Q136–NADPH hydrogen bond and (ii) populating eclipsed substrate conformations involving the migrating methyl.^8^ For AHB turnover, however, the loss of the Q136–NADPH hydrogen bond was not expected to confer a catalytic advantage over WT, because WT-AHB already showed weak Q136–NADPH hydrogen bonding as previously stated. Additionally, the migrating methyl in ACL is substituted with a migrating ethyl in AHB, and near-eclipsed substrate conformations were associated with WT turnover efficiency for ACL but not AHB as shown previously. To determine whether S487A-AHB may have lost a catalytic advantage over WT-AHB with respect to migrating ethyl torsional orientation, we analyzed the dihedral angle (*Dihe AC6/O6,AC6/C4,AC6/C5,AC6/C33*), tracking the alignment of the migrating ethyl group to other substrate atoms (i.e., assessing the degree of eclipsing). The distribution of this torsion differed between S487A-AHB and WT-AHB. While the WT-AHB ethyl flexibly sampled eclipsed and staggered conformations, the S487A-AHB ethyl was largely restricted to the two staggered conformations near −60° and 60°, and it did not sample the near-staggered conformation around −150° to −170° that was associated with successful turnover in WT-AHB (Figure 9A). Notably, the staggered conformation near −60° was unique to S487A-AHB across all variants (Figure S4). The rarity of this conformation, coupled with its high density of NR_–0.4_ trajectories relative to R ones, suggested that it was unfavorable for catalysis of AHB.

**Figure 9:**
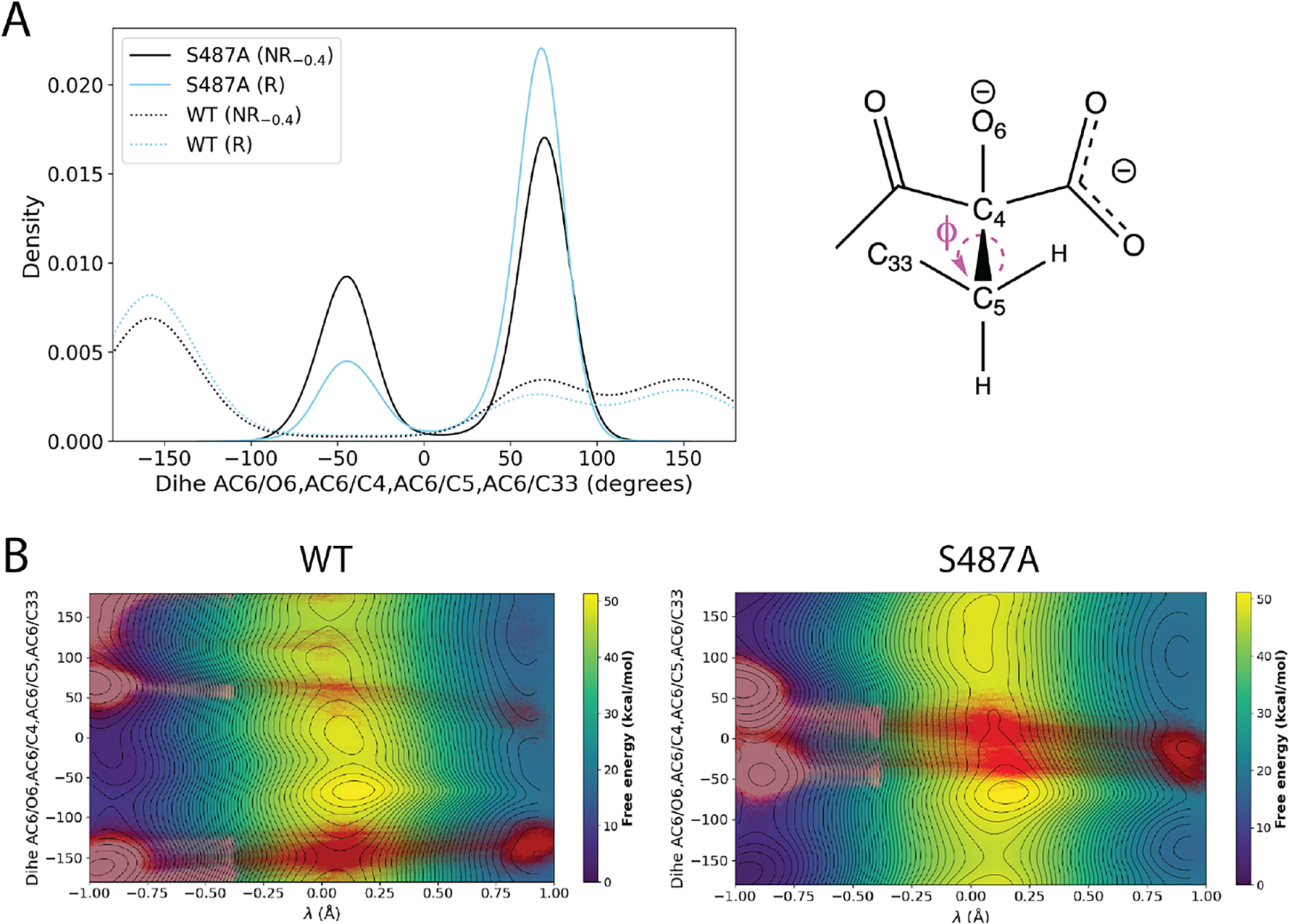
S487A-AHB catalysis. (A) Distribution of the dihedral angle describing AHB ethyl conformation within the −160 to −130 fs time window for reactive (R, blue) and nonreactive (NR_–0.4_, black) trajectories sampled by WT-AHB (dashed) and S487A-AHB (solid). (B) Two-dimensional PMF surface (free energy) along the order parameter λ (*x*-axis) and the dihedral angle describing the migrating ethyl conformation (*y*-axis) for WT-AHB (left) and S487A-AHB (right). Red and pink lines designate R and NR_–0.4_ trajectories, respectively.

We next analyzed the effect of the migrating ethyl conformation on the reaction energy by computing a two-dimensional PMF (potential of mean force; free energy) surface along the order parameter, λ, and the dihedral angle using umbrella sampling with WHAM (weighted histogram analysis method; Figure 9B). Starting with WT-AHB, reaction attempts typically initiated from energy wells near staggered conformations, with the exception of the metastable −60° staggered conformation. The conformations at 60° and 180° were only separated by a barrier of 3 kcal/mol, which may explain the flexibility with which WT-AHB sampled conformations within this region. Reaction attempts from the eclipsed 120° conformation were also observed. Overall, the most densely populated reaction channel, which was also the most catalytically efficient, sampled conformations slightly on the negative side of the 180° staggered conformation (i.e., −150° to −180°). These conformations accessed the product well via a channel with the lowest peak in free energy relative to other sampled channels. Overall, WT-AHB initiated reactions from multiple migrating ethyl conformations in the reactant well, and attempted reactions could reach and enter the product well via multiple routes sampled by different ethyl conformations. Moving now to S487A-AHB, the sampled space of migrating ethyl conformations was more restricted. S487A-AHB only sampled the two staggered conformations near 60° and −60°, the latter of which corresponded with a shallow energy well that was not identified for WT-AHB. While reactions were attempted from both conformations, the ethyl typically shifted toward 0° on the approach to reacting, and successful attempts always entered the product well with the ethyl around 0° to −50°. Similar to WT-AHB, an energetically favorable channel was identified near −150° to −180° for S487A-AHB. However, S487A-AHB never sampled reactant well conformations with the ethyl in the corresponding staggered conformation needed to access this channel. The catalytically inefficient −50° conformation had a higher peak free energy along its path toward the product well, but it also had a higher ground state (GS) energy such that its Δ*G* ^‡^ was lower compared to the 60° conformation. Thus, the difference in efficiency between these two conformations cannot be attributed to Δ*G* ^‡^ alone.

In summary, S487A had increased activity on ACL by weakening the Q136–NADPH hydrogen bond and increasing the population of eclipsed substrate conformations involving the migrating methyl, compared to WT. For catalysis of AHB, however, the weakening of the Q136–NADPH hydrogen bond was not expected to affect reaction rate, and the conformational dynamics of S487A-AHB’s migrating ethyl were not consistent with efficient, reactive-like conformations sampled by WT-AHB. Instead, the torsional orientation of the substrate migrating ethyl in S487A-AHB was more consistent with failed reaction attempts and could thereby explain S487A’s low activity on AHB relative to WT.

### Unique Mg^2+^ Coordination Identified in M472Q-AHB Associated with Reduced *k*_cat_

Like L501H and S487A, M472Q catalyzed ACL more efficiently than WT by weakening the Q136–NADPH hydrogen bond and increasing the population of eclipsed substrate conformations involving the migrating methyl in ACL. As shown for L501H and S487A, these structural changes relative to WT were conducive to ACL turnover but not AHB turnover, and calculated activity of the mutants on AHB was similar to or lower than WT on AHB. Thus, WT-like activity on AHB may be viewed as an upper limit for M472Q, but we wanted to determine why M472Q-AHB had a *k*_cat_ more than 300-fold slower than that for WT-AHB. The R and NR_–0.4_ pathways from M472Q-AHB were distributed across two groups in the UMAP projection (Figure 6). One of the groups at UMAP Axis 1 > 10 was found to be unique with respect to the behavior of its Mg^2+^-coordinating waters. Specifically, O_21_, which typically coordinated M_16_, instead coordinated M_17_ in these trajectories (Figure 10A). The coordination of M_17_ by O_21_ was not observed in other KARI-substrate complexes (Figure S5), which suggested that it could be unfavorable for catalysis of ACL and AHB. The two-dimensional PMF (free energy) surface along the order parameter and a coordinate tracking the difference in the distances from O_21_ to M_16_ and M_17_ measured a 1.4–2.9 kcal/mol higher Δ*G* ^‡^ for conformations with M_17_ coordinated by O_21_ compared to the more common coordination arrangement (Figure 10B). Therefore, M472Q-AHB may have lower activity than WT-AHB due to the larger reaction barrier for its sometimes altered Mg^2+^ coordination, which was unique to M472Q-AHB. The perturbed Mg^2+^ coordination geometry was more accessible to M472Q-AHB than WT-AHB. That is, the activation energy to transition from M_16_–O_21_ coordination to M_17_–O_21_ coordination was ≈2 kcal/mol lower for M472Q-AHB than for WT-AHB (Figure 10B). The ≈3 kcal/mol barrier between these two conformations in M472Q-AHB was still large enough that all reaction attempts were stably in one conformation or the other before, during, and after attempting to react (red and pink lines, Figure 10B).

**Figure 10:**
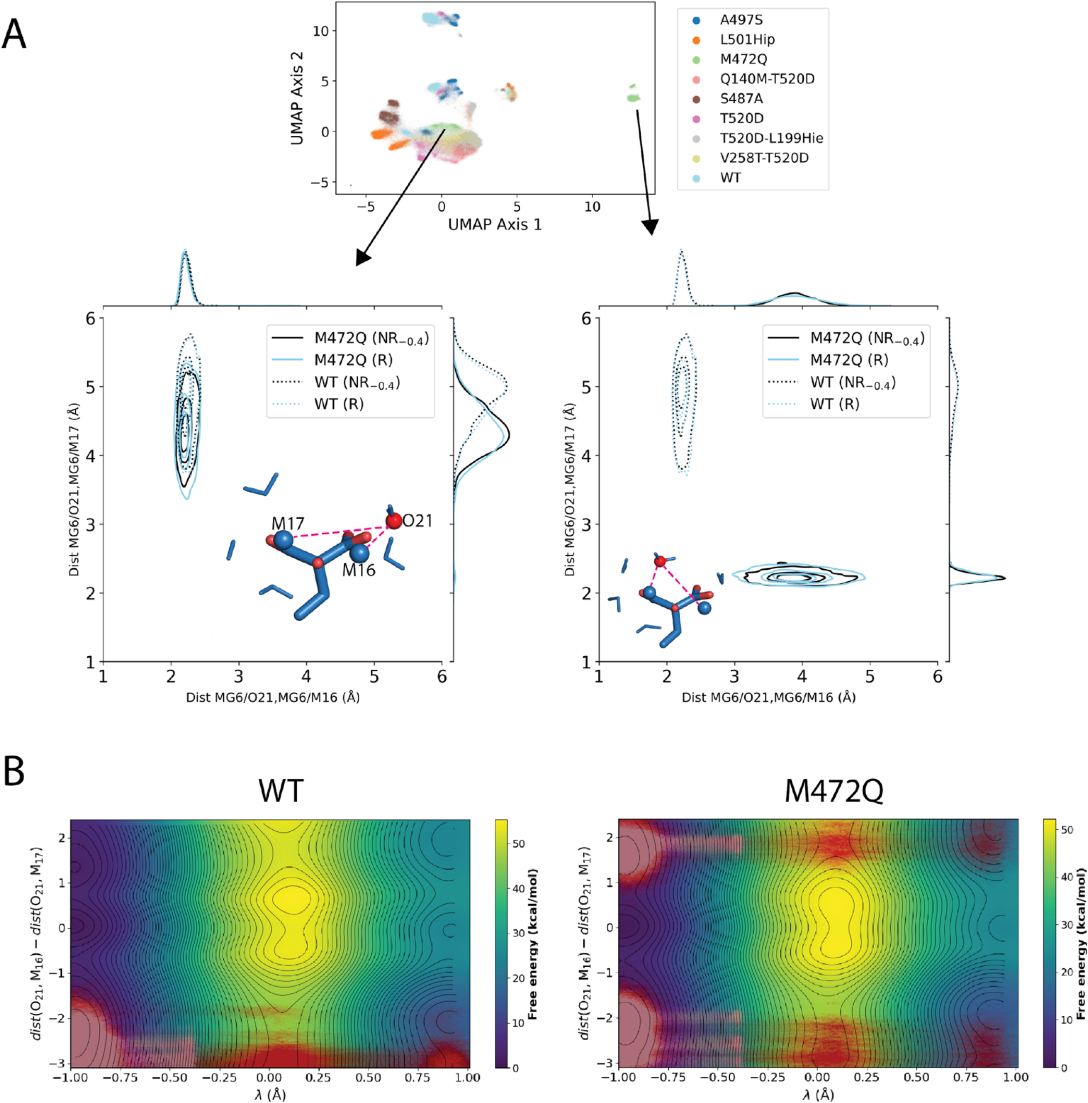
M472Q-AHB catalysis. (A) Distributions of the distances from MG6/O_21_ to MG6/M_16_ (*x*-axis) and MG6/M_17_ (*y*-axis) for R (blue) and NR_–0.4_ (black) trajectories sampled by WT-AHB (dashed) M472Q-AHB (solid). The M472Q-AHB trajectories from the two UMAP groups (arrows) were separately plotted to clearly indicate their difference in O_21_ coordination. (B) Two-dimensional PMF surface (free energy) along the order parameter λ (*x*-axis) and a coordinate tracking the difference in the distances from O_21_ to M_16_ and M_17_ (in Å, *y*-axis) for WT-AHB (left) and M472Q-AHB (right). Large, positive *y*-axis values correspond with closer coordination of M_17_ than M_16_ by O_21_. Red and pink lines designate R and NR_–0.4_ trajectories, respectively.

Despite the increased Δ*G* ^‡^ associated with M_17_–O_21_ coordination relative to M_16_–O_21_ coordination, the probability densities of R and NR_–0.4_ trajectories were similar. This observation implies that the catalytic efficiency was unaffected by the altered coordination, which is at odds with its increased Δ*G* ^‡^. It appears that increased Δ*G* ^‡^, alone, did not fully determine reaction efficiency and that there were other, perhaps kinetic, factors playing a role as well.

## Conclusion

In an earlier study, we identified eight KARI mutants with increased computed activity on ACL relative to WT, and the increased catalytic efficiencies were associated with specific, quantifiable structural changes near the active site, most notably (i) the loss of a hydrogen bond between Q136 and the amide group of NADPH’s nicotinamide, (ii) the rotation of E319’s carboxylate, and (iii) the increased sampling of near-eclipsed substrate conformations involving the migrating methyl. Most of the identified mutants seemed to increase activity on ACL, relative to WT, by way of activating two out of these three reaction-facilitating structural changes. Here, we tested how these mutations, designed for enhanced activity on ACL, affected catalysis on KARI’s other native substrate, AHB. If there was strong evolutionary pressure for increased specific activity on both substrates, one might expect the fact that evolution has not selected these mutants for spinach KARI to mean that they decrease specific activity on AHB. This was found to be the case for only two out of the eight mutants using TIS rate constant calculations. Of the remaining six mutants, three had significantly larger (i.e., greater than 10-fold) computed *k*_cat_ for AHB than WT, and three were within an order of magnitude. The spread in mutant activities on AHB suggested that perhaps only some of the structural changes associated with increased ACL turnover efficiency translated to catalysis of AHB, and it ruled out the notion that these ACL reaction-promoting mutations were uncommon among KARI enzyme sequences because they detrimentally affected catalysis of AHB, the other native substrate.

Using a methodology that paralleled the TIS rate constant calculations, atomistic simulations of attempted reactions by the enzyme were sampled. Subsequent analyses of reactive (R) and nonreactive (NR_–0.4_) trajectories suggested that of the three structural features associated with improved ACL turnover, only (ii) the rotated E319 conformation was expected to directly translate to, and facilitate, AHB catalysis. The Q136–NADPH hydrogen bond (i) did not associate with R vs NR_–0.4_ WT-AHB trajectories while the nature of eclipsed substrate conformations (iii) was different for ACL- and AHB-bound enzymes because of their different migrating alkyl groups.

All four of the AHB-bound T520D-containing mutants sampled the rotated E319 conformation, and all four had computed *k*_cat,AHB_ that were at least as large as WT’s, with three of the four posting values that were increased over WT’s by more than 100-fold. Of the three mutants shown to enhance ACL turnover efficiency by structural changes (i) the weakening of the Q136–NADPH hydrogen bond and (iii) the sampling of eclipsed substrate conformations, two had computed *k*_cat,AHB_ more than 100-fold smaller than WT’s while the other was within 1 order of magnitude. These *k*_cat,AHB_ values were consistent with the notion that only (ii) the rotated E319 structural change enhanced turnover of AHB similar to its effect on ACL. Further structural analysis of reaction trajectories and free energy calculations implicated catalytically inefficient migrating ethyl conformations as a potential cause for S487A-AHB’s limited activity, and similar analyses and calculations identified a conformation with altered Mg^2+^-water coordination and an increased reaction barrier (i.e., Δ*G* ^‡^), which was unique to M472Q-AHB and may thereby explain its low *k*_cat_.

Interestingly, we observed first that a collection of mutants that increased KARI’s computed *k*_cat_ on ACL operated by a variety of mechanisms, and also that only a subset of those mechanisms transferred computationally to the substrate AHB. Taken together, it is possible to design mutants predicted to increase activity against one substrate while making directed changes in specificity for a second.

## Supplementary Material


