# Supplementary material for: Substrate-Specific Differences in Catalytic Strategy and Activity across Ketol-Acid Reductoisomerase Variants
Source: J Phys Chem B. 2026 Jul 15;130(30):7562–77. doi: 10.1021/acs.jpcb.6c03868 (PMC13430628; doi:10.1021/acs.jpcb.6c03868)
Supplement: Supplementary file 1 [file jp6c03868_si_001.pdf]

Substrate-Specific Differences in Catalytic Strategy and Activity across Ketol-Acid Reductoisomerase Variants (Supporting Information)

Elijah Karvelis[†,‡] and Bruce Tidor[*,†,‡,¶]

†*Department of Biological Engineering, Massachusetts Institute of Technology, Cambridge, MA 02139, USA*

‡*Computer Science and Artificial Intelligence Laboratory, Massachusetts Institute of Technology, Cambridge, MA 02139, USA*

¶*Department of Electrical Engineering and Computer Science, Massachusetts Institute of Technology, Cambridge, MA 02139, USA*

E-mail: tidor@mit.edu

Table S1: TIS-computed k_{cat} (s^{-1}) for ACL and AHB alkyl transfer.

Variant	ACL	AHB
WT	$(9.5 \pm 3.9) \times 10^{-17}$	$(1.5 \pm 1.0) \times 10^{-14}$
Q140M-T520D	$(1.7 \pm 1.4) \times 10^{-12}$	$(6.6 \pm 6.6) \times 10^{-10}$
T520D-L199H	$(8.6 \pm 3.4) \times 10^{-15}$	$(1.0 \pm 0.6) \times 10^{-11}$
V258T-T520D	$(6.1 \pm 3.0) \times 10^{-15}$	$(6.7 \pm 5.5) \times 10^{-12}$
T520D	$(8.5 \pm 3.5) \times 10^{-15}$	$(1.1 \pm 0.9) \times 10^{-13}$
L501H	$(1.8 \pm 1.5) \times 10^{-13}$	$(3.2 \pm 1.4) \times 10^{-14}$
A497S	$(6.6 \pm 1.5) \times 10^{-16}$	$(2.4 \pm 1.0) \times 10^{-14}$
M472Q	$(5.4 \pm 3.7) \times 10^{-15}$	$(4.2 \pm 2.1) \times 10^{-17}$
S487A	$(8.5 \pm 4.7) \times 10^{-15}$	$(9.4 \pm 5.4) \times 10^{-18}$

Values reported as AVG $\pm$ SEM, $n = 9$ independent TIS rate constant calculations.

Table S2: Computed TIS flux factor, Φ_A (fs^{-1}), for ACL and AHB alkyl transfer.

Variant	ACL	AHB
WT	$(6.3 \pm 0.2) \times 10^{-4}$	$(8.6 \pm 0.4) \times 10^{-5}$
Q140M-T520D	$(1.47 \pm 0.03) \times 10^{-3}$	$(6.6 \pm 0.3) \times 10^{-4}$
T520D-L199H	$(1.36 \pm 0.04) \times 10^{-3}$	$(5.9 \pm 0.3) \times 10^{-4}$
V258T-T520D	$(1.03 \pm 0.02) \times 10^{-3}$	$(3.6 \pm 0.3) \times 10^{-4}$
T520D	$(1.00 \pm 0.03) \times 10^{-3}$	$(5.1 \pm 0.4) \times 10^{-4}$
L501H	$(1.13 \pm 0.05) \times 10^{-3}$	$(2.7 \pm 0.1) \times 10^{-4}$
A497S	$(9.6 \pm 0.1) \times 10^{-4}$	$(2.3 \pm 0.1) \times 10^{-4}$
M472Q	$(9.0 \pm 0.3) \times 10^{-4}$	$(1.6 \pm 0.1) \times 10^{-4}$
S487A	$(9.6 \pm 0.1) \times 10^{-4}$	$(1.3 \pm 0.1) \times 10^{-4}$

Values reported as $\text{AVG} \pm \text{SEM}, n = 9$ independent calculations.

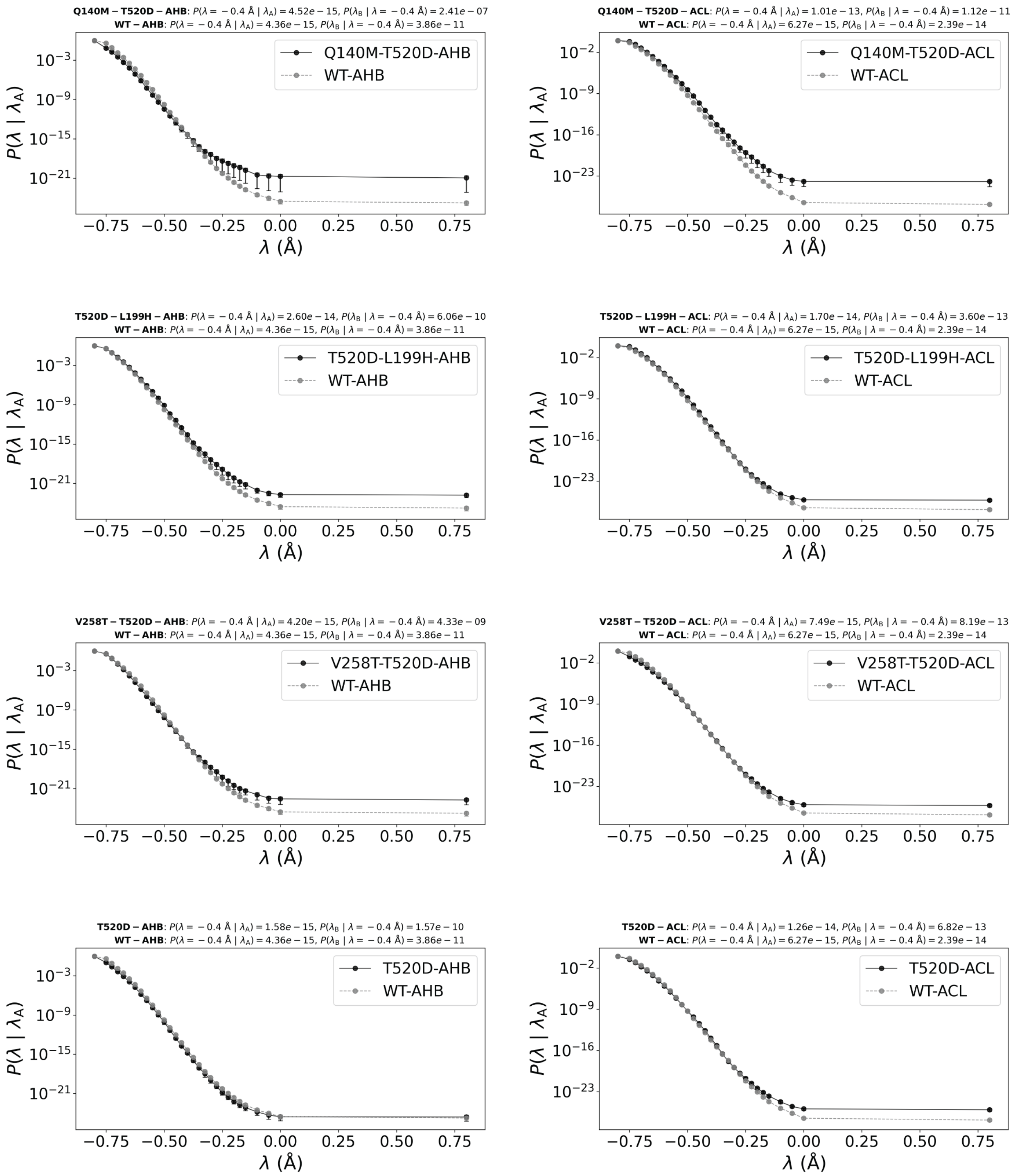
Q140M − T520D − AHB: P(λ = −0.4 Å | λ_A) = 4.52e − 15, P(λ_B | λ = −0.4 Å) = 2.41e − 07
WT − AHB: P(λ = −0.4 Å | λ_A) = 4.36e − 15, P(λ_B | λ = −0.4 Å) = 3.86e − 11
Q140M-T520D-AHB
WT-AHB
Q140M − T520D − ACL: P(λ = −0.4 Å | λ_A) = 1.01e − 13, P(λ_B | λ = −0.4 Å) = 1.12e − 11
WT − ACL: P(λ = −0.4 Å | λ_A) = 6.27e − 15, P(λ_B | λ = −0.4 Å) = 2.39e − 14
Q140M-T520D-ACL
WT-ACL
T520D − L199H − AHB: P(λ = −0.4 Å | λ_A) = 2.60e − 14, P(λ_B | λ = −0.4 Å) = 6.06e − 10
WT − AHB: P(λ = −0.4 Å | λ_A) = 4.36e − 15, P(λ_B | λ = −0.4 Å) = 3.86e − 11
T520D-L199H-AHB
WT-AHB
T520D − L199H − ACL: P(λ = −0.4 Å | λ_A) = 1.70e − 14, P(λ_B | λ = −0.4 Å) = 3.60e − 13
WT − ACL: P(λ = −0.4 Å | λ_A) = 6.27e − 15, P(λ_B | λ = −0.4 Å) = 2.39e − 14
T520D-L199H-ACL
WT-ACL
V258T − T520D − AHB: P(λ = −0.4 Å | λ_A) = 4.20e − 15, P(λ_B | λ = −0.4 Å) = 4.33e − 09
WT − AHB: P(λ = −0.4 Å | λ_A) = 4.36e − 15, P(λ_B | λ = −0.4 Å) = 3.86e − 11
V258T-T520D-AHB
WT-AHB
V258T − T520D − ACL: P(λ = −0.4 Å | λ_A) = 7.49e − 15, P(λ_B | λ = −0.4 Å) = 8.19e − 13
WT − ACL: P(λ = −0.4 Å | λ_A) = 6.27e − 15, P(λ_B | λ = −0.4 Å) = 2.39e − 14
V258T-T520D-ACL
WT-ACL
T520D − AHB: P(λ = −0.4 Å | λ_A) = 1.58e − 15, P(λ_B | λ = −0.4 Å) = 1.57e − 10
WT − AHB: P(λ = −0.4 Å | λ_A) = 4.36e − 15, P(λ_B | λ = −0.4 Å) = 3.86e − 11
T520D-AHB
WT-AHB
T520D − ACL: P(λ = −0.4 Å | λ_A) = 1.26e − 14, P(λ_B | λ = −0.4 Å) = 6.82e − 13
WT − ACL: P(λ = −0.4 Å | λ_A) = 6.27e − 15, P(λ_B | λ = −0.4 Å) = 2.39e − 14
T520D-ACL
WT-ACL
P(λ | λ_A)
λ (Å)

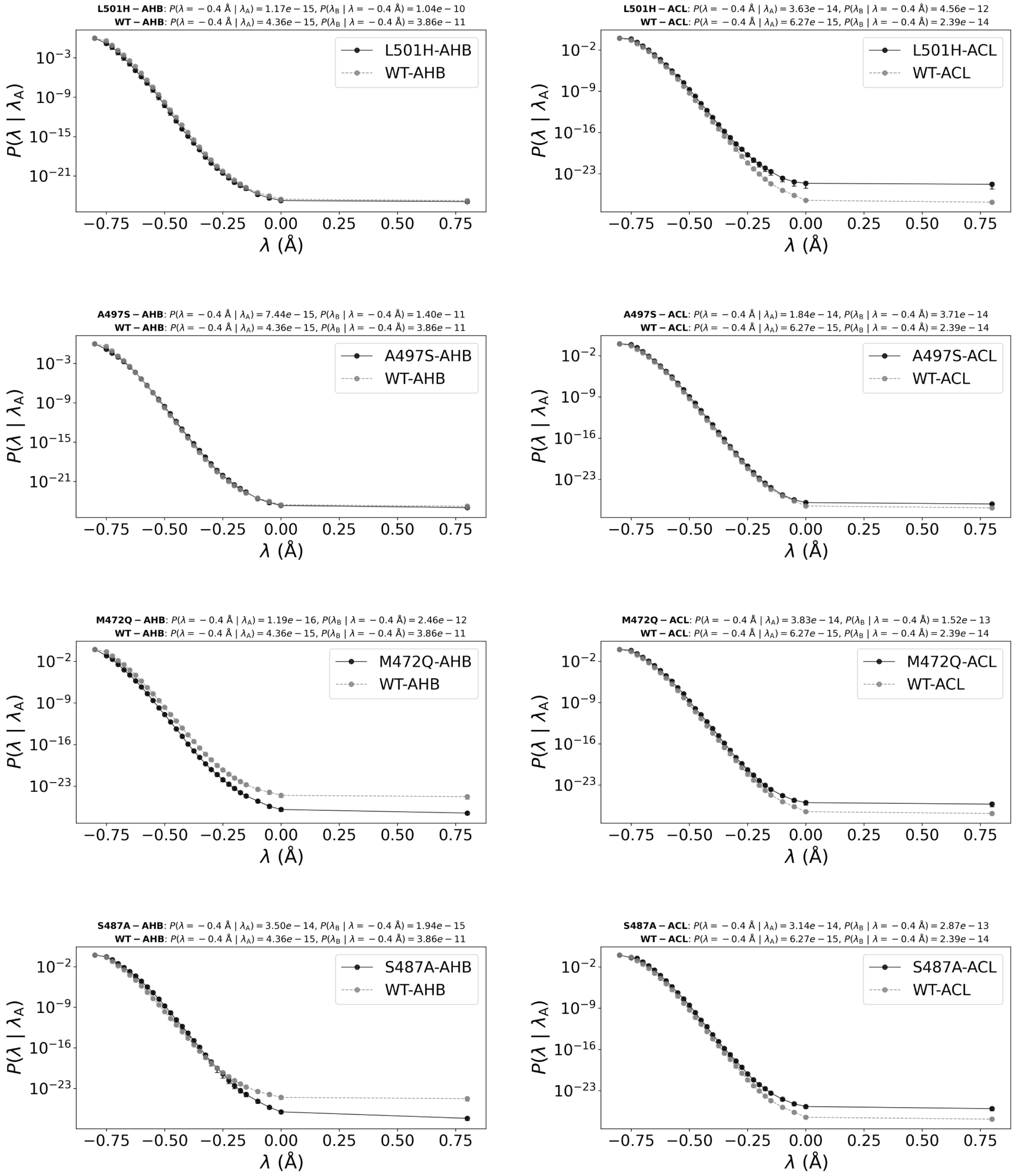

Figure S1: Cumulative kinetic probability plots shown here for mutant (solid) and WT (dashed) catalysis of AHB (left) and ACL (right).

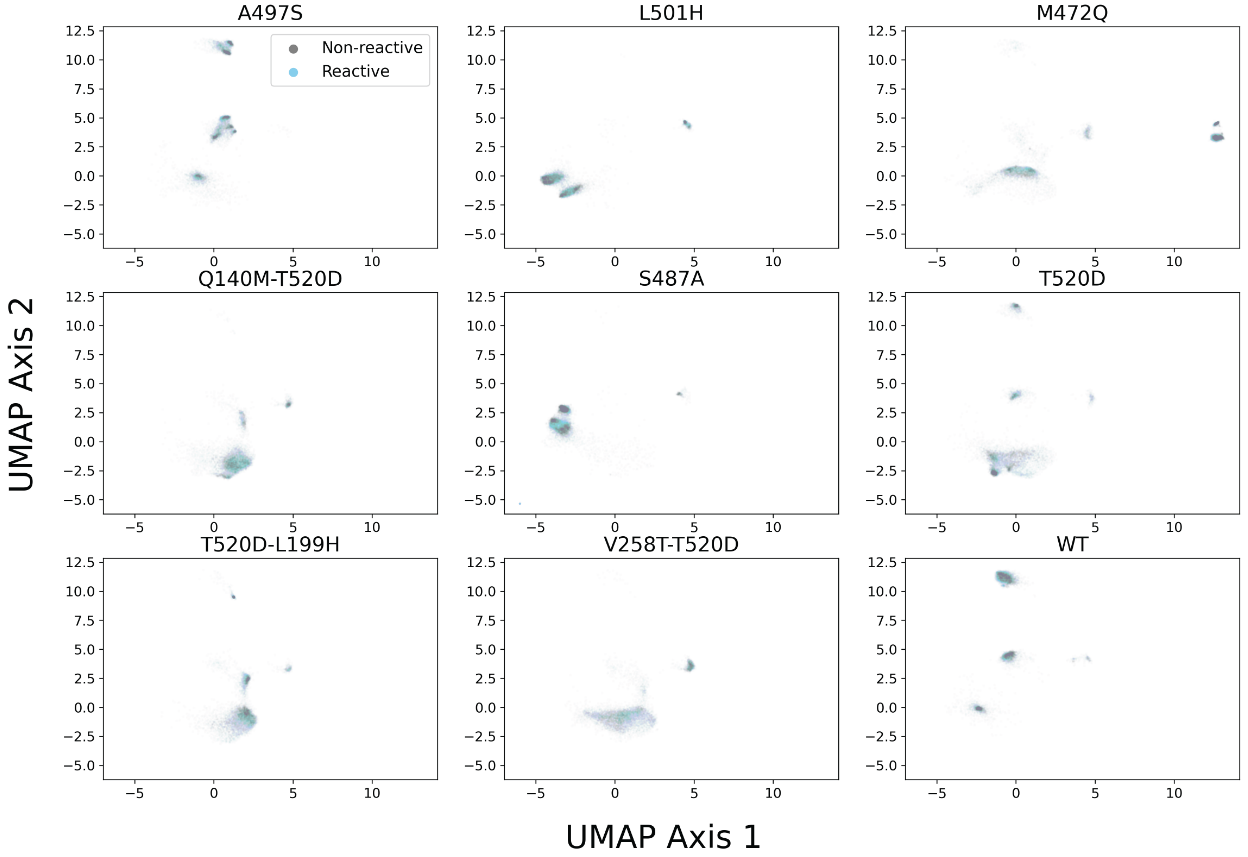

Figure S2: UMAP embedding of structural features describing enzyme-substrate complexes from KARI-AHB trajectories colored by pathway type (gray, $NR_{-0.4}$; blue, R) and shown for each variant on separate axes. The embedded conformations were randomly sampled from the -160 to -130 fs time window.

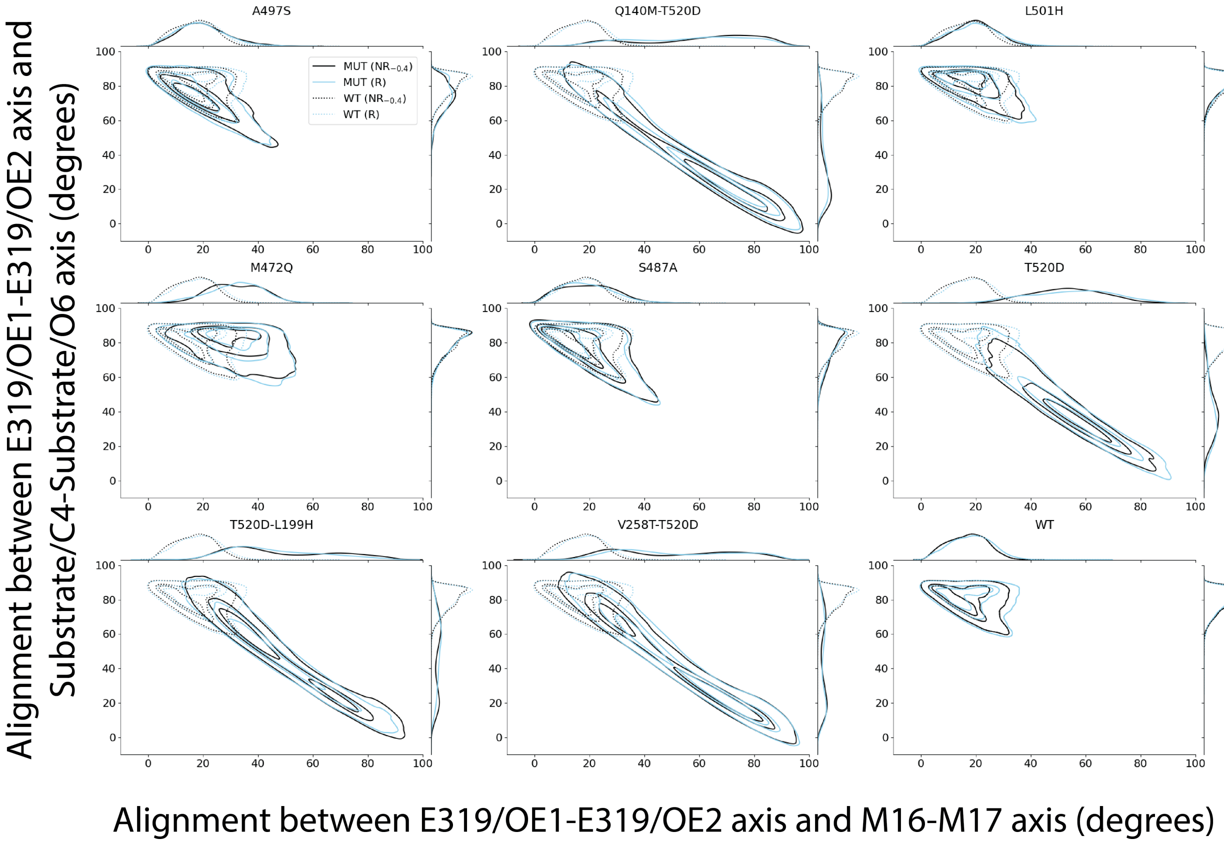

Figure S3: Tracking E319 conformation in KARI-AHB variants. Two-dimensional distributions over the angles (in degrees) between the axis joining the E319 carboxylate oxygens and the axis joining the Mg^{2+} cofactors (x-axis) or joining C_4 and O_6 (y-axis) for WT-AHB (dashed) and mutant-AHB (solid) R (blue) and $NR_{-0.4}$ (black) pathways. In the upper left, the axis spanning the E319 carboxylate oxygens is more closely aligned with the axis joining the Mg^{2+} cofactors. In the lower right, the axis spanning the E319 carboxylate oxygens is instead more closely aligned with the axis joining C_4 and O_6, which is referred to as a "rotated E319" conformation.

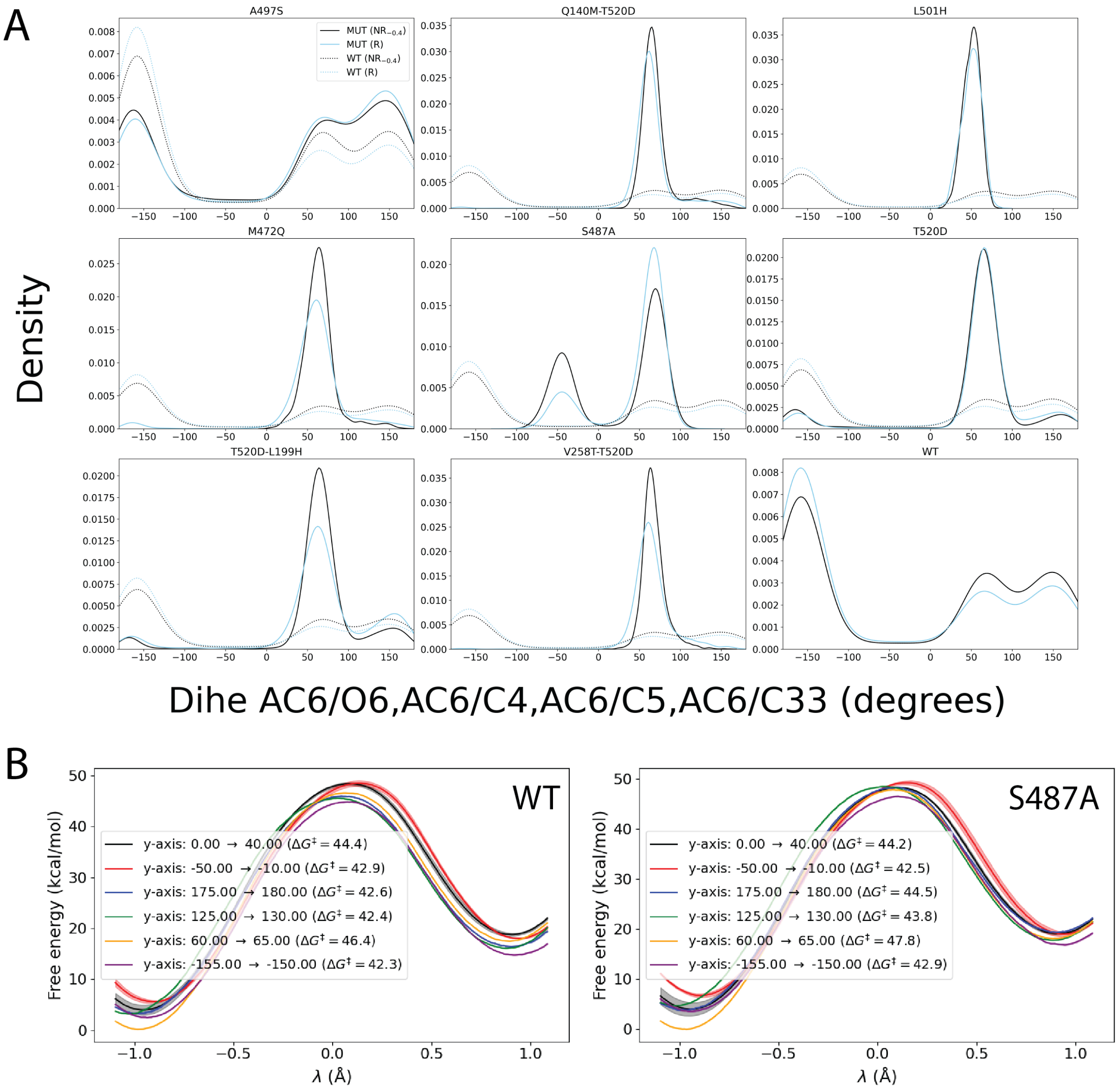

Figure S4: S487A-AHB catalysis. (A) Distribution of the dihedral angle describing AHB ethyl conformation (in degrees) within the -160 to -130 fs time window for reactive (R, blue) and non-reactive ($NR_{-0.4}$, black) trajectories sampled by WT-AHB (dashed) and mutant-AHB (solid). (B) One-dimensional cross sections taken within the indicated y-axis ranges from the two-dimensional PMF surfaces (free energy) in Figure 9B, which plot the free energy (kcal/mol) over the order parameter λ (in Å, x-axis) and the dihedral angle describing the migrating ethyl conformation (in degrees, y-axis). Left, WT-AHB; right, S487A-AHB. Lines track the average and shading indicates $\pm$ SD across all computed y-axis coordinates within the indicated range.

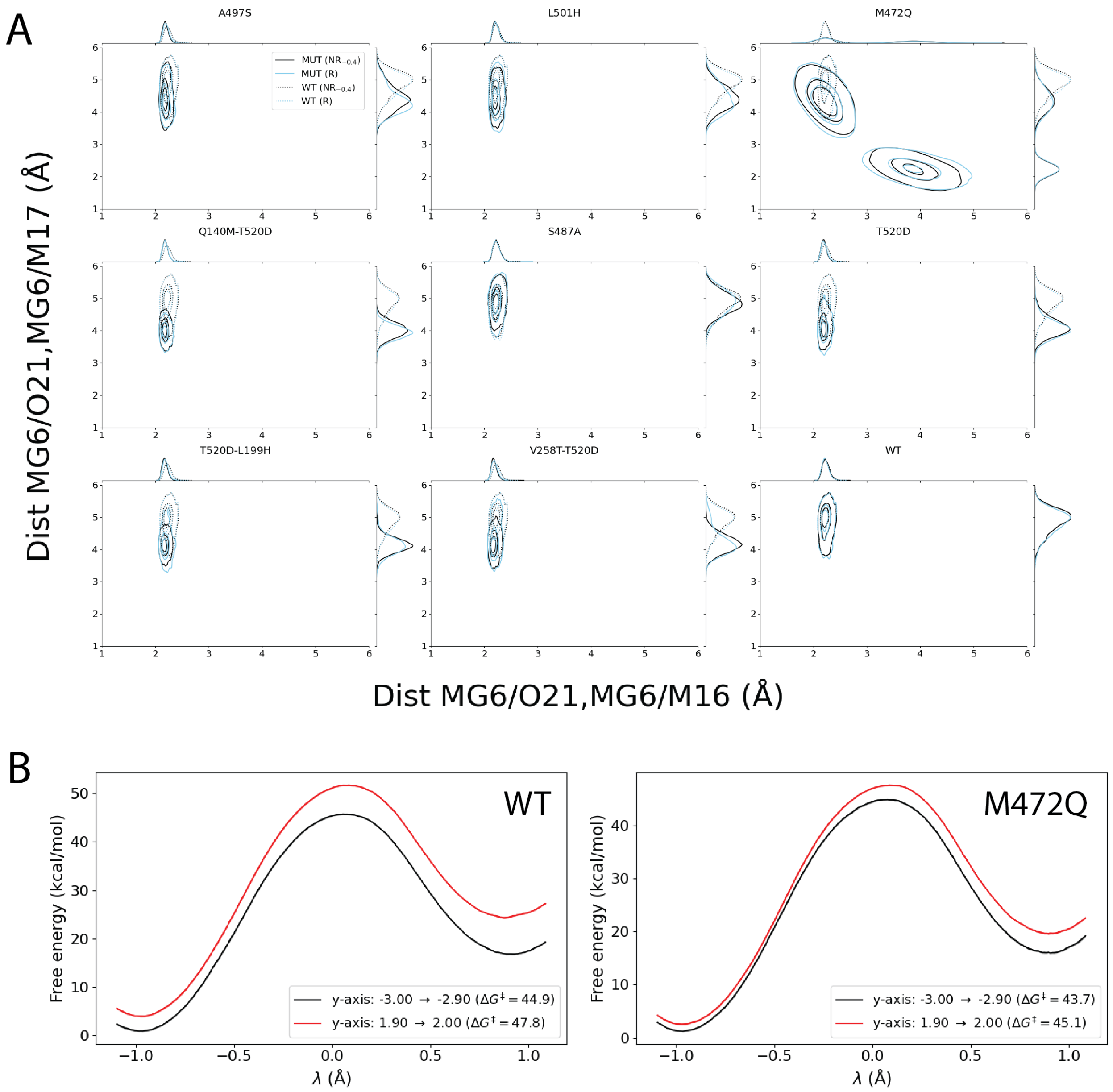

Figure S5: M472Q-AHB catalysis. (A) Distributions of the distances from MG6/O21$_{21}$ to MG6/M$_{16}$ (x-axis) and MG6/M$_{17}$ (y-axis) for R (blue) and NR$_{-0.4}$ (black) trajectories sampled by WT-AHB (dashed) and mutant-AHB (solid). (B) One-dimensional cross sections taken within the indicated y-axis ranges from the two-dimensional PMF surfaces (free energy) in Figure 10B, which plot the free energy (kcal/mol) over the order parameter λ (in Å, x-axis) and a coordinate tracking the difference in the distances from O_{21} to M_{16} and M_{17} (in Å, y-axis). Large, positive y-axis values correspond with closer coordination of M_{17} than M_{16} by O_{21}. Left, WT-AHB; right, M472Q-AHB. Lines track the average and shading indicates $\pm$ SD across all computed y-axis coordinates within the indicated range.

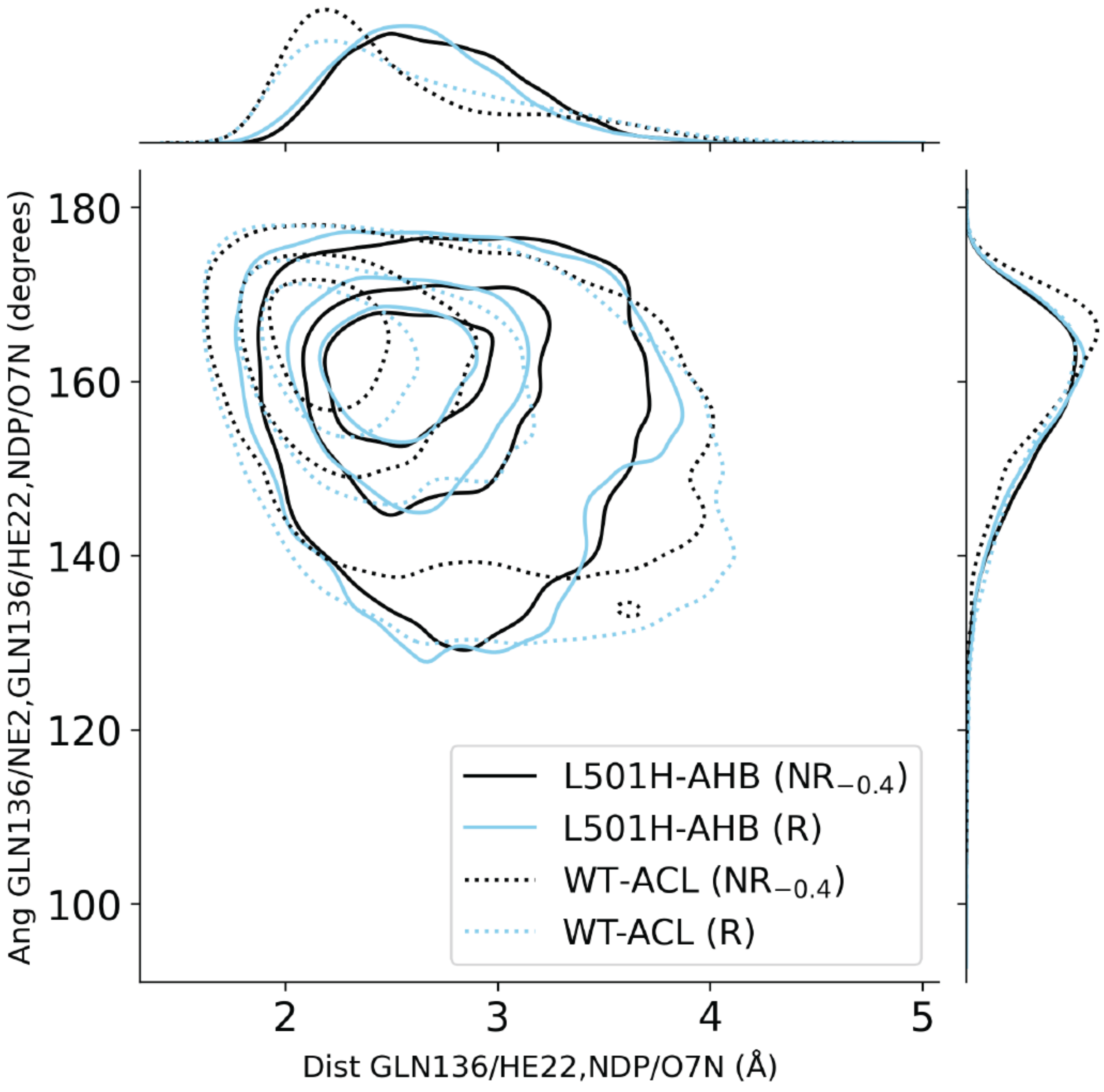

Figure S6: Q136 – NADPH hydrogen bonding in L501H-AHB and WT-ACL. Two-dimensional distribution over Q136 – NADPH hydrogen bond distance (x-axis) and angle (y-axis) within the −160 to −130 fs time window for reactive (R, blue) and non-reactive (NR$_{-0.4}$, black) trajectories sampled by WT-ACL (dashed) and L501H-AHB (solid).
